# Implementation of functional imaging using ^11^C-methionine PET-CT co-registered with MRI for advanced surgical planning and decision making in prolactinoma surgery

**DOI:** 10.1007/s11102-022-01230-2

**Published:** 2022-05-26

**Authors:** Leontine E. H. Bakker, Marco J. T. Verstegen, Eidrees Ghariq, Berit M. Verbist, Pieter J. Schutte, Waiel A. Bashari, Mark C. Kruit, Alberto M. Pereira, Mark Gurnell, Nienke R. Biermasz, Wouter R. van Furth, Lenka M. Pereira Arias Bouda

**Affiliations:** 1 Division of Endocrinology, Department of Medicine, Leiden University Medical Center, Leiden, The Netherlands; 2 Center for Endocrine Tumors Leiden (CETL), Pituitary Center, Leiden University Medical Center, Leiden, The Netherlands; 3 Department of Neurosurgery, Leiden University Medical Center, Leiden, The Netherlands; 4 Section of Nuclear Medicine, Department of Radiology, Leiden University Medical Center, Leiden, The Netherlands; 5 Department of Radiology, Leiden University Medical Center, Leiden, The Netherlands; 6 Wellcome–MRC Institute of Metabolic Science, Addenbrooke’s Hospital, University of Cambridge and National Institute for Health Research Cambridge Biomedical Research Centre, Cambridge Biomedical Campus, Cambridge, CB2 0QQ UK

**Keywords:** Prolactinoma, Functional imaging, Positron emission tomography, Surgical decision making, Transsphenoidal surgery, Dopamine agonist intolerance

## Abstract

**Purpose:**

To report the first experience of our multidisciplinary team with functional imaging using ^11^C-methionine positron emission tomography-computed tomography (^11^C-methionine PET-CT) co-registered with MRI (Met-PET/MRI^CR^) in clinical decision making and surgical planning of patients with difficult to treat prolactinoma.

**Methods:**

In eighteen patients with prolactinoma, referred to our tertiary referral centre because of intolerance or resistance for dopamine agonists (DA), Met-PET/MRI^CR^ was used to aid decision-making regarding therapy.

**Results:**

Met-PET/MRI^CR^ was positive in 94% of the patients. MRI and Met-PET/MRI^CR^ findings were completely concordant in five patients, partially concordant in nine patients, and non-concordant in four patients. In five patients Met-PET/MRI^CR^ identified lesion(s) that were retrospectively also visible on MRI. Met-PET/MRI^CR^ was false negative in one patient, with a cystic adenoma on conventional MRI. Thirteen patients underwent transsphenoidal surgery, with nine achieving full biochemical remission, two clinical improvement and near normalized prolactin levels, and one patient clinical improvement with significant tumour reduction. Hence, nearly all patients (94%) were considered to have a positive outcome. Permanent complication rate was low. Three patients continued DA, two patients have a wait and scan policy.

**Conclusion:**

Met-PET/MRI^CR^ can provide additional information to guide multidisciplinary preoperative and intraoperative decision making in selected cases of prolactinoma. This approach resulted in a high remission rate with a low rate of complications in our expert centre.

**Supplementary Information:**

The online version contains supplementary material available at 10.1007/s11102-022-01230-2.

## Introduction

Prolactinomas account for approximately 40% of all pituitary tumours [1]. Hyperprolactinemia leads to hypogonadism, subfertility, galactorrhoea and decreased bone density, and more general symptoms such as fatigue and psychological problems, resulting in decreased health-related quality of life (HR-QoL) [2–4].

Current standard care is medical treatment with dopamine agonists (DA), but long-term remission rates are lower than previously suggested and some patients experience intolerable mental or physical side effects. Surgery has traditionally been considered a rescue treatment for patients with drug intolerance, resistance, or mass-related complications. Recently, surgical indications for prolactinoma have been revisited, particularly for those tumours amenable to gross total resection (GTR) [5]. If GTR can be pursued safely and without significant risk of causing permanent pituitary hormone deficiencies, surgery may be preferable to mid to long-term DA use in some patients. According to a recent systematic literature review, when imaging reveals a distinct tumour, the surgical approach yields good outcomes at a group level and appears to be more cost-effective compared to medical treatment [2]. However, optimal pre-surgical counselling and planning of these cases is mandatory to achieve the highest standards of care and best possible outcomes. This is particularly true for difficult remnants or recurrences after prior medical or surgical treatment.

For small prolactinoma (remnants) and those in close proximity to the cavernous sinus, conventional imaging has limitations to reliably identify the precise localization and invasion of surrounding structures, and to discriminate between normal gland and adenoma. Hence, in order to improve the prediction of chances for remission and to minimize surgically induced pituitary insufficiency there is an unmet need for better tumour localization. Therefore, we initiated a structural collaboration with the Cambridge pituitary multidisciplinary team, which has developed and optimized functional imaging using ^11^C-methionine to enable more accurate localization of functioning pituitary adenomas. This tracer is preferentially taken up by the adenoma and to a lesser extent by the normal pituitary with relatively low uptake by the surrounding brain. Co-registration of ^11^C-methionine positron emission tomography-computed tomography with MRI (Met-PET/MRI^CR^) allows the site of maximal ^11^C-methionine uptake to be more accurately defined and addresses the important question of whether a possible lesion detected on structural imaging coincides with functional activity.

To date, data correlating imaging findings with subsequent treatment decisions and clinical outcomes in patients with functioning pituitary adenomas, in particular in prolactinoma, is very limited. The Cambridge group has reported clear added value of Met-PET/MRI^CR^ for treatment decisions in selected patients with Cushing’s disease and persistent acromegaly after primary therapy [6–9]. The applicability of this type of diagnostics requires a well-equipped multidisciplinary team, know-how, and experience. Similar studies using MRI co-registration and correlating imaging findings with clinical decision making and outcome have not yet been performed in patients with prolactinomas.

Therefore, the aim of this study is to report the experience of our multidisciplinary pituitary team with this diagnostic tool newly implemented in our centre. Specifically, we have examined its role in clinical decision making and surgical planning of 18 patients with prolactinoma referred to our tertiary centre because of intolerance or resistance to medical treatment with DA, and to describe how treatment decisions were influenced by the findings on Met-PET/MRI^CR^.

## Methodology

### Description of centre and care path

The Pituitary Centre of the Leiden University Medical Centre is a tertiary referral centre for pituitary and complex endoscopic skull base surgery, performing over 150 surgeries a year. There is a dedicated multidisciplinary team (MDT), with 3 neurosurgeons, 3 endocrinologists, 2 neuroradiologists, 2 nuclear medicine physicians, an ophthalmologist, a radiation oncologist, a psychologist and a nurse specialist case manager, that have meetings every second week. All patients are cared for in a predefined pituitary care pathway as described previously and comprehensive outcomes are collected prospectively with their informed consent (G19.011) [10].

### Description of subjects

Prospective approval was obtained from the Dutch Health and Youth Inspectorate for clinical implementation of Met-PET/CT in patients with complex pituitary disease. The institutional review board approved this study and informed consent was obtained from all patients as described above. Met-PET/MRI^CR^ was performed between 7–2018 and 8–2020 in 18 patients with prolactinoma (13 women, 5 men; median age 31 years, range 18–59 years; 6 micro-, 12 macro-prolactinoma (for patient characteristics see: Tables 1, 2 and Online Resource 2 for a table with detailed characteristics per patient)). All patients had been managed in accordance with local and international clinical guidelines, but usually the referral letter included a request for surgical counselling. The decision to perform a Met-PET/MRI^CR^ was undertaken on a case-by-case basis following discussion in our local pituitary MDT. If indicated, patients were also discussed in a second international pituitary MDT meeting together with the Cambridge group, e.g. to share experience in very difficult cases, to learn from their experience with the technique and to discuss imaging findings obtained from Met-PET/MRI^CR^. The Cambridge-Leiden MDT comprised pituitary neurosurgeons, endocrinologists, and nuclear medicine physicians of both centres. Treatment decisions based on MRI alone, and on MRI plus Met-PET/MRI^CR^, both as discussed in our local MDT and as discussed in the Cambridge-Leiden MDT, were reported. Furthermore, in our local MDT we estimate the chance of remission and risks in advance and assess for complications afterwards (two weeks and six months postoperatively). All consultation steps were assessed in a protocolized and structured manner. This paper reports all 18 consecutively made Met-PET/MRI^CR^ scans in prolactinoma patients.

**Table 1:** Clinical and biochemical characteristics of all patients together and per group

	All	Group 1	Group 2	Group 3
Number of patients	18	4	8	6
Age (years)	31 (18–59)	31 (19–51)	29 (18–59)	31 (27–53)
Sex (female: male)	13:5	3:1	4:4	6:0
Duration of disease (months)	89 (6–408)	53 (6–316)	100 (12–408)	97 (28–194)
Prolactin at diagnosis (ULN)	7.2 (2.5–208)	4.0 (2.5–6.0)	22.0 (7.2–208)	6.4 (4.0–48)
Prolactin at Met-PET/MRI^CR^ (ULN)	4.6 (1.4–161)	3.5 (2.5–4.6)	13.8 (1.4–161)	4.3 (2.4–5.5)
Micro: Macroadenoma	6:12	2:2	1:7	3:3
Prior surgery	9	2	4	3
Prior medication	18	4	8	6
Duration of prior medical treatment (months)	66 (2–403)	46 (2–216)	60 (7–403)	73 (12–91)

Data are presented as median (min–max) or number

*ULN* upper limit of normal

**Table 2:** Radiological characteristics and outcome of all patients together and per group

	All	Group 1	Group 2	Group 3
Initial pre-treatment MRI available	11/18	3/4	3/8	5/6
Initial size (mm)	14 (5–37)	10.0 (7–12)	36.0 (28–37)	11.5 (5–20)
CS invasion initial MRI Knosp 3B-4	5/11 3/11	1/3 0/3	3/3 3/3	1/5 0/5
CS invasion last MRI before Met-PET Knosp 3B-4	9/18 yes, 2/18 possibly 5/18	2/4 0/4	5/8 yes, 2/8 possibly 5/8	2/6 yes 0/6
Remnant size (mm)	6.5 (3–25)	6.5 (6–13)	13.5 (5–25)	4.5 (3–7)
CS invasion Met-PET/MRI^CR^	9/18 yes, 1/18 uncertain	2/4	5/8 yes, 1/8 uncertain	2/6 yes
Estimated remission chance at baseline	3.0 (1–5) (2.67 ± 1.24)	4.5 (2–5)	1.5 (1–3)	3.0 (2–3)
Estimated remission chance after	4.0 (1–5)* (3.33 ± 1.41)*	5.0 (5–5)	2.5 (1–4)*	3.5 (2–4)
Estimated complication risk at baseline	2.0 (1–3) (1.89 ± 0.76)	1.5 (1–2)	2.0 (1–3)	2.0 (1–3)
Estimated complication risk after	1.5 (1–3) (1.72 ± 0.83)	1.0 (1–2)	2.0 (1–3)	1.0 (1–3)
MDT Cambridge	6/18	0/4	4/8	2/6
Surgery	13/18	4/4	5/8	4/6
Confirmative histopathology	10/13 1 uncertain	3/4	5/5	2/4, 1 uncertain
Biochemical remission	9/13	4/4	4/5	1/4
Clinical improvement	13/13	4/4	5/5	3/4
Complications	1/13 permanent 6/13 temporary	0/4 permanent 1/4 temporary	1/5 permanent 2/5 temporary	0/4 permanent 3/4 temporary

Data are presented as median (min–max) or number of total number. For estimated remission chances and complication risks also mean ± SEM is reported in brackets for the whole group. *CS* cavernous sinus, *MDT* multi-disciplinary team. The Wilcoxon signed-rank test was used to compare the estimated remission chance and complication risk before and after Met-PET/MRI^CR^

*p < 0.05

### Patient groups and diagnostic dilemmas

All referred patients had received prior treatment before inclusion, DA only (n = 9 patients) and DA plus surgery (n = 9 patients). Most patients experienced persistent symptomatic hyperprolactinemia and intolerance for DA. Based on the clinical situation and the radiological adenoma characteristics, we identified typical diagnostic dilemmas and patients were classified accordingly in the following groups:

1. Met-PET/MRI^CR^ for confirmation (4 subjects): prolactinoma with a highly suspected (remnant) lesion on MRI. In these cases, with high a priori chance of remission and low a priori risk of complications, Met-PET/MRI^CR^ was acquired as extra supporting assessment that the lesion was highly feasible for total resection, and to provide the patient with a better estimate of the likelihood of reaching the desired goals.
2. Met-PET/MRI^CR^ for additional information (8 subjects): difficult remnant after surgery and/or longstanding medical treatment with indeterminate findings on conventional MRI (e.g. unclear remnant, uncertainty about extension, possible multifocality and/or relation with the cavernous sinus) in patients with a high need for alternative treatment because of DA intolerance and/or resistance. Functional imaging was used to obtain additional information for a more accurate benefit-risk ratio of treatment options: (i) to visualize the exact location(s) and extension of the remnant, (ii) to discriminate between post-treatment change (e.g. sella remodelling due to scar tissue) and residual functional adenoma, or (iii) to assess the proportion of most active remnant tissue that could be safely removed with targeted debulking surgery or treated with targeted radiotherapy (i.e. Met-PET/MRI^CR^ guided therapy).
3. Met-PET/MRI^CR^ for diagnosis (6 subjects): no (clear) adenoma visible on conventional MRI. Functional imaging was used to localize a remnant that was not clearly visible on MRI from diagnosis, or to explore remnants of an initially visible adenoma that were less clearly visible as a result of surgery and/or medical therapy-induced shrinkage.

Because these groups have differences in characteristics, medical need, a priori chances and outcomes, the groups are described separately and we refrain from summarizing outcomes of the total group. We report on the decision-making process and, where available, outcomes are also reported.

### Outcomes

The intended effect was defined as (1) biochemical remission, defined as normalization of the prolactin level within 3 months postoperatively without the need for adjunctive medical therapy, or (2) debulking as planned with clinically significant tumour reduction. As a secondary outcome, clinical improvement was recorded, defined as improved prolactin levels with or without lower doses of DA and clinical improvement of the patient, e.g. restoration of menstrual cycle. Adverse effects were defined as (1) all clinically relevant complications [e.g. cerebrospinal fluid (CSF) leakage, reoperation because of a bleeding, CSF leakage or epistaxis, readmission for hyponatremia or other causes, meningitis, transient and permanent pituitary insufficiency including diabetes insipidus (DI)], and as (2) permanent complications (e.g. persistent DI and/or anterior pituitary hormone deficiency, persistent nerve damage).

* A priori estimation of chances for remission and complications:* Before and after Met-PET/MRI^CR^ an estimate was made of the chance of remission and risks by the neurosurgeon and endocrinologist. The estimated chance of remission was divided into 5 classes: very unlikely (~ 0 to ~ 20%), unlikely (~ 21 to ~ 40%), possibly (~ 41 to ~ 60%), likely (~ 61 to ~ 80%), and very likely (~ 81 to ~ 100%). The estimated chance of long-term risks, mainly determined by the risk of permanent pituitary deficiencies, was divided into 3 classes: low (< ~ 2%), moderate (~ 2 to ~ 5%), and high (> ~ 5%).

### Imaging techniques

#### MRI

All MR imaging acquired at our institution was performed on an Achieva 3.0 T TXMR system (Philips Healthcare, Best, The Netherlands) using a commercial 32-channel head coil, according to our local MR pituitary protocol. On initial presentation the protocol consisted of coronal and sagittal T1-weighted, turbo spin echo (T1w TSE, in-plane resolution 0.45 × 0.56 mm and 0.47 × 0.59 mm respectively, with a slice thickness of 3 mm), and coronal T2 weighted, TSE (T2w TSE, resolution 0.36 × 0.40 mm, slice thickness 3 mm) imaging of the sella region before intravenous (i.v.) contrast administration, dynamic coronal T1 weighted fast field echo (T1w FFE, resolution 1.02 × 1.02 mm, slice thickness 1.5 mm) imaging of the sella during i.v. administration of 0.1 mmol/kg gadoterate meglumine (gd-DOTA, DOTAREM; Guerbet, Roissy‐Charles de Gaulle Cedex, France), and coronal and sagittal T1w TSE imaging of the sella after i.v. contrast administration. On follow-up imaging no pre-contrast sagittal T1w TSE and coronal dynamic T1w FFE images were acquired. In addition, when appropriate (i.e. before ^11^C-methionine PET imaging for registration purposes) an additional post-contrast axial 3D T1w FFE with whole brain coverage was acquired (resolution 1 × 1 mm, 1 mm slice thickness) in the same frame of reference as the pituitary sequences.

#### PET-CT imaging with ^11^C-methionine (Met-PET/CT)

Patients were instructed to fast for at least 6 h prior to the PET imaging. l-[methyl-^11^C]-methionine (^11^C-methionine), was synthesized in compliance with good manufacturing practice at the Radionuclide Centre of the Amsterdam University Medical Centre (Amsterdam UMC) location VU University Medical Centre (VUMC), Amsterdam, the Netherlands, as described previously [11]. PET-CT scans were acquired at the department of Radiology and Nuclear medicine of the VUMC 20 min after injection of a 5 MBq/kg bolus of ^11^C-methionine intravenously. PET scan duration was 20 min. Prior to each PET scan a low dose computed tomography (ldCT, 220 mAs, 140 kV, 0.5 s rotation, 0.984 mm pitch, 1.25 mm slice thickness) scan of the entire head was acquired for attenuation and scatter correction purposes. PET images were then iteratively reconstructed with attenuation correction using a 3D reconstruction algorithm with a 2 mm Gaussian smoothing filter and 2 mm slice thickness.

#### Image processing and co-registration with MRI (Met-PET/MRI^CR^)

Image processing and co-registration with MRI were performed in our hospital, using IntelliSpace Portal version 10 (ISP, Philips Healthcare). First, all the data were transferred to a dedicated system with ISP. Met-PET and MRI images were 3D co-registered using a normalised mutual information-based automatic registration algorithm. For this purpose, the 3D T1w FFE MRI sequence was selected as the primary dataset. The ldCT dataset acquired as part of the Met-PET-CT imaging was then co-registered with the MRI and the resulting registration parameters were applied to the PET data to achieve a Met-PET/MRI^CR^ registration. The accuracy of the co-registration was then visually examined by checking the degree of agreement between certain landmarks with (significant) ^11^C-methionine uptake, i.e. lacrimal glands, cerebral cortex and pons.

Lastly, the PET methionine uptake maps were individually thresholded to the corresponding (background) cerebellar uptake to discern adenoma uptake from normal pituitary uptake. The registered MR and PET images were read into the multimodality viewing software of ISP, which overlays the PET onto the MRI and produces fused as well as source images in the same (coronal, sagittal and axial) planes.

All MRI images were independently reviewed by experienced neuroradiologists on dedicated PACS workstations using Sectra IDS7 software (Sectra Imtec AB, Linköping, Sweden) without a knowledge of the PET findings. The absence or presence of cavernous sinus invasion was defined according to Knosp criteria [12, 13]. Finally, all the images were reviewed by an experienced nuclear medicine physician and discussed in a multidisciplinary team as described above.

### Statistics

We report descriptive statistics. Data are presented as median (minimum–maximum). The Wilcoxon signed-rank test was used to compare the estimated remission chance and complication risk before and after Met-PET/MRI^CR^. Significance level was set at p < 0.05. Statistical analyses were performed using SPSS for Windows version 26.0 (IBM, USA).

## Results

### Overall performance of Met-PET/MRI^CR^ in prolactinoma patients

In 17 of 18 (94%) patients Met-PET/MRI^CR^ identified one or more foci of increased tracer uptake either confirming suspicious areas identified on MRI or revealing previously unsuspected sites of residual adenoma. One patient (subject 13) had a cystic adenoma which showed no uptake of methionine on Met-PET/MRI^CR^. MRI and Met-PET/MRI^CR^ were completely concordant in 5 patients (subjects 6, 12, 9, 15, 18), partially concordant in 9 patients (1, 2, 3, 4, 7, 8, 10, 16, 17), and non-concordant in 4 patients (subjects 5, 11, 13, 14). In 5 patients the Met-PET identified lesion(s) were retrospectively also visible on MRI-scan (subjects 3, 4, 8, 10, 17).

### Group decision process and outcomes

See Online Resource 1, 2 and 3 for all case descriptions, table and figures.

### Group 1: Met-PET/MRI^CR^ for confirmation (subjects 4, 13, 15 (Fig. 1) and 18 (Fig. 1))

**Fig. 1:**
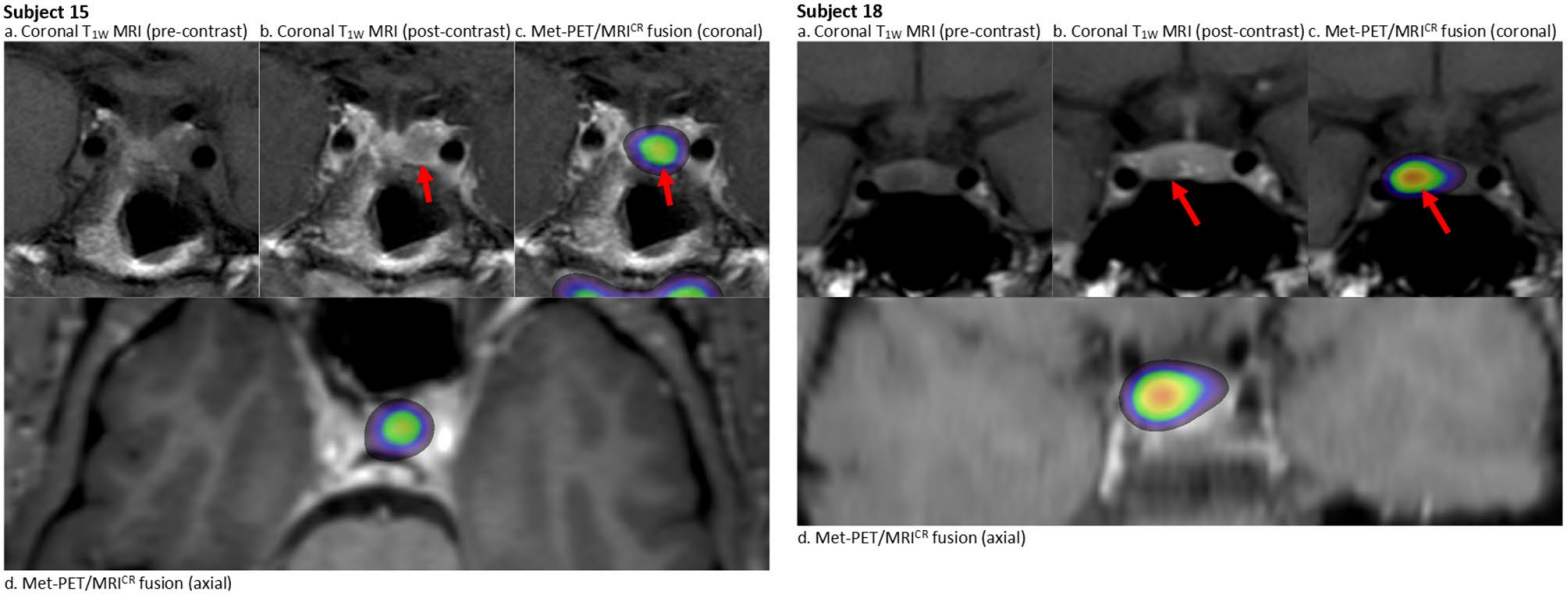
Imaging in subjects 15 and 18 (group 1: Met-PET/MRI^CR^ for confirmation). Subject 15: Post-surgical MRI of the pituitary gland coronal pre- (**a**) and post-contrast (**b**) T1 weighted images (T1w) and ^11^C-methionine PET images overlayed on MRI images (coronal (**c**) and axial (**d**) post-contrast T1 weighted images). The red arrow indicates the increased methionine uptake (SUV ratio of 2.95) compared to cerebellum in the hypo-enhancing soft tissue on the left confirming active for adenoma. Subject 18: MRI of the pituitary gland coronal pre- (**a**) and post-contrast (**b**) T1 weighted images (T1w) and ^11^C-methionine PET images overlayed on MRI images (coronal (**c**) and axial (**d**) post-contrast T1 weighted images). The red arrow indicates the focus with the highest methionine uptake (SUV ratio of 3.0) compared to cerebellum which corresponded to a hypo-enhancing soft tissue compared to the rest of the sellar tissue

In four patients MRI showed a highly suspected (remnant) adenoma. All four had been treated with DA for a variable duration of time but were experiencing significant side effects, and two subjects had also been operated previously. In surgery naïve subjects 13 and 18 the estimated likelihood of remission and complication risk were already very likely and low, respectively, on MRI at baseline, which did not change substantially after Met-PET/MRI^CR^. Met-PET/MRI^CR^ was mainly performed to obtain the highest possible certainty in these elective cases for prolactinoma surgery. These cases allowed the multidisciplinary team to gain experience with this mode of imaging in the early days of Met-PET/MRI^CR^ use and to confirm applicability in patients with prolactinomas. In subject 13, with a cystic lesion on MRI, Met-PET/MRI^CR^ showed no uptake of methionine. In subject 18, with a well-defined right-sided intrasellar adenoma without cavernous sinus invasion (CSI) on MRI, Met-PET/MRI^CR^ was completely in concordance. Both subjects underwent TSS with intraoperative findings concordant to (MR) imaging. Both patients obtained biochemical remission, and no complications occurred. Histopathological confirmation was obtained in one patient (subject 18), while in the other predominant staining for prolactin and growth hormone (GH) was present without clear proof of adenoma or pituitary gland tissue (subject 13). In previously DA treated and operated subjects 4 and 15, Met-PET/MRI^CR^ was used to discriminate between postoperative remodelling and residual adenoma again to obtain more certainty before proceeding to surgery, and showed clear increased tracer uptake in one specific site in both patients concordant with MRI. Estimated likelihood of remission changed from likely to very likely with an unchanged estimated moderate risk of complications (subject 4), and from unlikely to very likely with a decreased risk of complications from moderate to low (subject 15). Both underwent TSS with perioperative findings in concordance with imaging, positive histopathology and normalization of prolactin within one week. Subject 4 experienced transient DI.

### Group 2: Met-PET/MRI^CR^ for additional information (subjects 1, 2, 3, 6 (Fig. 2), 9 (Fig. 2), 12, 16 and 17 (Fig. 2))

**Fig. 2:**
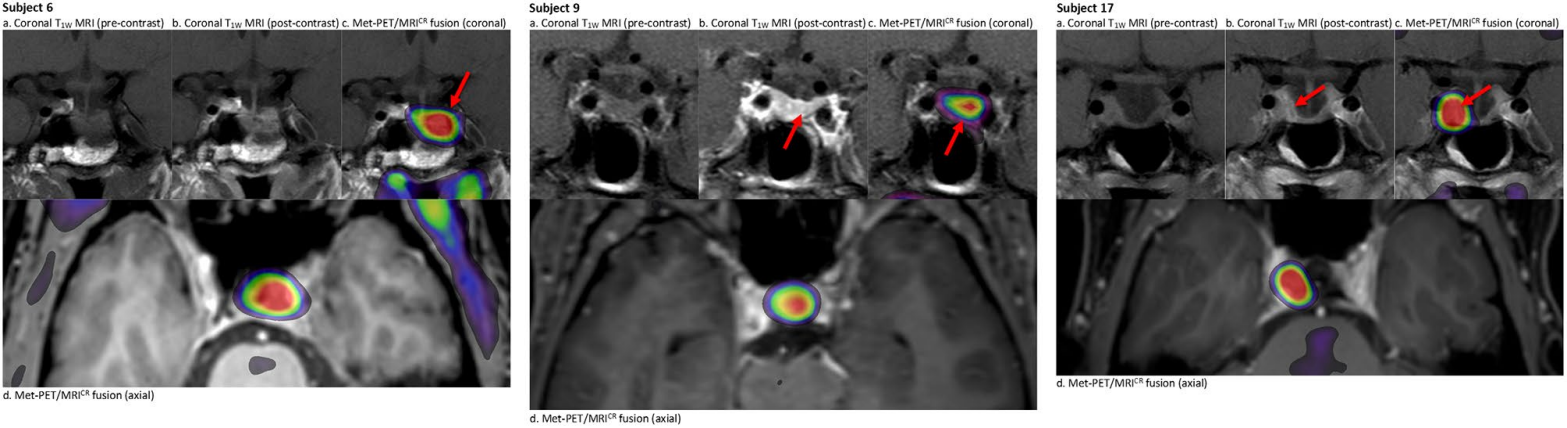
Imaging in subjects 6, 9 and 17 (group 2: Met-PET/MRI^CR^ for additional information). Subject 6: MRI of the pituitary gland coronal pre- (**a**) and post-contrast (**b**) T1 weighted images (T1w) and ^11^C-methionine PET images overlayed on MRI images (coronal [c] and axial [d] post-contrast T1 weighted images). The red arrow indicates the focus with the highest methionine uptake (SUV ratio of 5.1) compared to cerebellum which corresponded to a hypo-enhancing soft tissue at the left side inside the sella. Subject 9: Post-surgical MRI of the pituitary gland coronal pre- (**a**) and post-contrast (**b**) T1 weighted images (T1w) and ^11^C-methionine PET images overlayed on MRI images (coronal [c] and axial [d] post-contrast T1 weighted images). The red arrow indicates the focus with the highest methionine uptake (SUV ratio of 3.46) compared to cerebellum which corresponded to a hypo-enhancing soft tissue at the left side intrasellar. Subject 17: Post-surgical MRI of the pituitary gland coronal pre- (**a**) and post-contrast (**b**) T1 weighted images (T1w) and ^11^C-methionine PET images overlayed on MRI images (coronal [c] and axial [d] post-contrast T1 weighted images). The red arrow indicates the focus with the highest methionine uptake (SUV ratio of 5.2) compared to cerebellum which corresponded to a rim of hypo-enhancing soft tissue medial to the right cavernous sinus without signs of invasion of the sinus on neither the MRI nor the PET

This group represents eight patients with difficult post-surgical remnants and/or longstanding medical treatment with indeterminate findings on conventional MRI (unclear remnant, uncertain extension, possible multifocality, uncertain CSI) in situations with a high need for an alternative treatment because of DA intolerance and/or resistance.

Three patients had macroprolactinoma remnants after previous treatment with uncertain CSI on MRI (subjects 1, 2 and 17). Subjects 2 and 17 had persistent disease despite medical and surgical treatment, and subject 1 had been treated with DA and was normoprolactinemic but was experiencing side effects. Estimated chances of remission based on MRI only were very unlikely (1 and 2) and possibly (17). Functional imaging was used to optimize preoperative ideas on surgical perspective. Remission chances remained very unlikely for subjects 1 and 2, but changed to likely in subject 17 (no signs for multifocality or CSI). Subsequently, subject 17 underwent surgery and obtained biochemical remission. Subject 1 underwent debulking surgery with significant reduction as anticipated with a concomitant decrease in prolactin level and relevant clinical improvement. The postoperative course was uncomplicated. On a low dose of cabergoline she became pregnant within three months postoperatively. For subject 2 radiotherapy was proposed, however, he elected to try lower DA dose, which he now tolerates, in contrast to the preoperative situation.

Three patients (subjects 3, 9, 16) had remnants of a macroprolactinoma after long standing medical treatment and/or surgery (subject 9, twice) with indeterminate findings on MRI, with suspicion for multifocality. Estimated remission chances were very unlikely (subject 16), and possibly (subjects 3 and 9). For subjects 3 and 9 functional imaging was used to localize the remnant, showing clear tracer uptake at one site in both subjects, increasing remission chances to likely. Surgery was performed with positive histopathology and normalization of prolactin within one week. For subject 16, Met-PET/MRI^CR^ was performed to explore surgical options. Although remission chances with TSS were still estimated to be unlikely with a high risk of complication, TSS was considered a realistic option for improving QoL given his high burden of disease after longstanding medical treatment. Nevertheless, the patient decided to continue with DA.

Subjects 6 and 12 had initially large adenomas with currently unclear extensions and anatomy after shrinkage due to treatment, and with a high need for adequate targeted treatment because of DA intolerance and/or resistance. In subject 6, functional imaging was performed to assess treatment options, changing the estimated chance of remission from unlikely to possibly. Given his intolerance and resistance to DA it was deemed reasonable to undertake surgical exploration. Quite unexpectedly, prolactin levels normalized. Subject 12 had prior debulking surgery because of regrowth while on DA despite previous shrinkage. Because of young age, drug side effects, and suspicion of an aggressive tumour, Met-PET/MRI^CR^ was performed to assess the possibilities of curative surgery and also to assess the most active part as a target for debulking surgery or radiotherapy. Estimated chance of remission remained very unlikely with a high risk for complications. Currently, we re-initiated medical therapy in an attempt to postpone the necessity for radiotherapy because surgery is not an option.

### Group 3: Met-PET/MRI^CR^ for diagnosis (subjects 5, 7 (Fig. 3), 8 (Fig. 3), 10 (Fig. 3), 11 and 14)

**Fig. 3:**
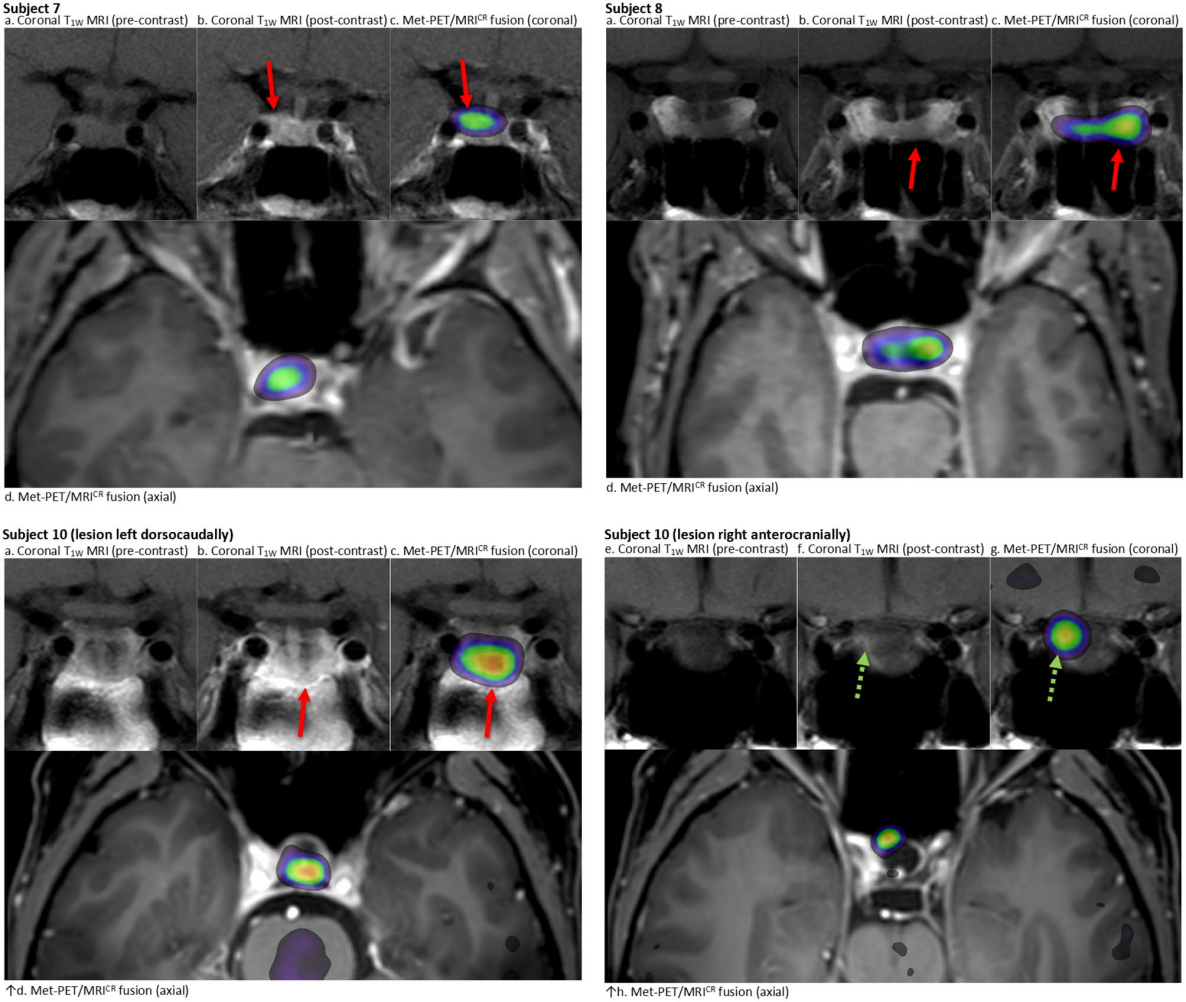
Imaging in subjects 7, 8 and 10 (group 3: Met-PET/MRI^CR^ for diagnosis). Subject 7: Post-surgical MRI of the pituitary gland coronal pre- (**a**) and post-contrast (**b**) T1 weighted images (T1w) and ^11^C-methionine PET images overlayed on MRI images (coronal (**c**) and axial (**d**) post-contrast T1 weighted images). The red arrow indicates the focus with the highest methionine uptake (maximum SUV ratio of 2.61) compared to cerebellum which corresponded to a hypo-enhancing soft tissue dorsolaterally intrasellar. Subject 8: MRI of the pituitary gland coronal pre- (**a**) and post-contrast (**b**) T1 weighted images (T1w) and ^11^C-methionine PET images overlayed on MRI images (coronal (**c**) and axial (**d**) post-contrast T1 weighted images). The red arrow indicates the focus with the highest methionine uptake (SUV ratio of 2.21) compared to cerebellum which corresponded to a hypo-enhancing soft tissue at the left inside the sella. Subject 10: Post-surgical MRI of the pituitary gland coronal pre- (**a** and** e**) and post-contrast (**b** and** f**) T1 weighted images (T1w) and ^11^C-methionine PET images overlayed on MRI images (coronal [c and g] and axial [d and h] post-contrast T1 weighted images). The red arrow indicates the focus with the highest methionine uptake (maximum SUV ratio of 2.81) compared to cerebellum which corresponded to a hypo-enhancing soft tissue dorsally inside the sella. The green dotted arrow indicates the second focus at the right anterolateral side of the sella, slightly more cranial than the first focus (maximum SUV ratio 2.71)

This group consists of six patients with no (clear) adenoma visible on conventional MRI, either from diagnosis or as a result of surgery and/or medically induced shrinkage.

In subjects 5, 7 and 11, even from the time of the initial diagnosis the prolactinoma could never be clearly discriminated from the normal pituitary gland on MRI. All three patients had been treated with DA, and all experienced marked side effects and had clear preference for surgery in an attempt to discontinue medical treatment. Subjects 5 and 7 had already been operated before with positive histopathology but without normalization of serum prolactin. Dedicated MRI-scans, including dynamic sequences, were inconclusive. Likelihoods for remission with TSS were estimated to be unlikely (subject 11) and possibly (subjects 5 and 7), with low to moderate complication risks. Met-PET/MRI^CR^ lowered the estimated remission chance to unlikely in subject 5, upon which we refrained from surgery. For subject 7, Met-PET/MRI^CR^ changed the remission chance to likely, showing clear tracer uptake on one site. She was operated for a third time and finally achieved biochemical remission. In subject 11, estimated chance of remission increased to possibly. She underwent surgery during which an apparently easy to remove firm adenoma on the sellar floor was completely resected, however histopathology was non-diagnostic. Her postoperative prolactin level did not decrease significantly although clinically she did experience improvement in her menstrual cycle and mental state, and recently became pregnant shortly after resuming a low dose of DA.

In subjects 8, 10 and 14, functional imaging was used to localize a remnant of an initially clearly visible prolactinoma that had shrunk after long standing medical treatment and/or surgery and was less clearly visible on current MRI. All three patients had been treated with DA with accompanying side effects with a treatment duration of 72–74 months, and one patient had also been operated (subject 10). Met-PET/MRI^CR^ showed tracer uptake on one site without CSI in two patients (subjects 8 and 14), increasing the likelihood of remission from possibly to likely, with estimated low risks. Surgery was performed with positive histopathology in both patients. Although normoprolactinemia was not obtained, both subjects did show significant clinical improvement [subject 8: postmenopausal, prolactin preoperatively 6.8 × ULN vs. 2.6 × ULN postoperatively (both without DA); subject 14: prolactin preoperatively 4.6 × ULN vs. 1.7 × ULN postoperatively with restoration of menstrual cycle]. Besides transient disturbances in fluid balance no complications occurred. In subject 10, Met-PET/MRI^CR^ showed two lesions, which were retrospectively also identified on previous MRI. Likelihood of remission remained possibly with a high estimated risk for complications due to previous surgery and medical treatment and localization of the pituitary gland. Given intolerance and resistance for DA, however, revision TSS was advised. Nevertheless, for now, the patient preferred a wait and scan policy without DA.

### Assessment of preoperative chances for remission and complications and impact of Met-PET/MRI^CR^

**Fig. 4:**
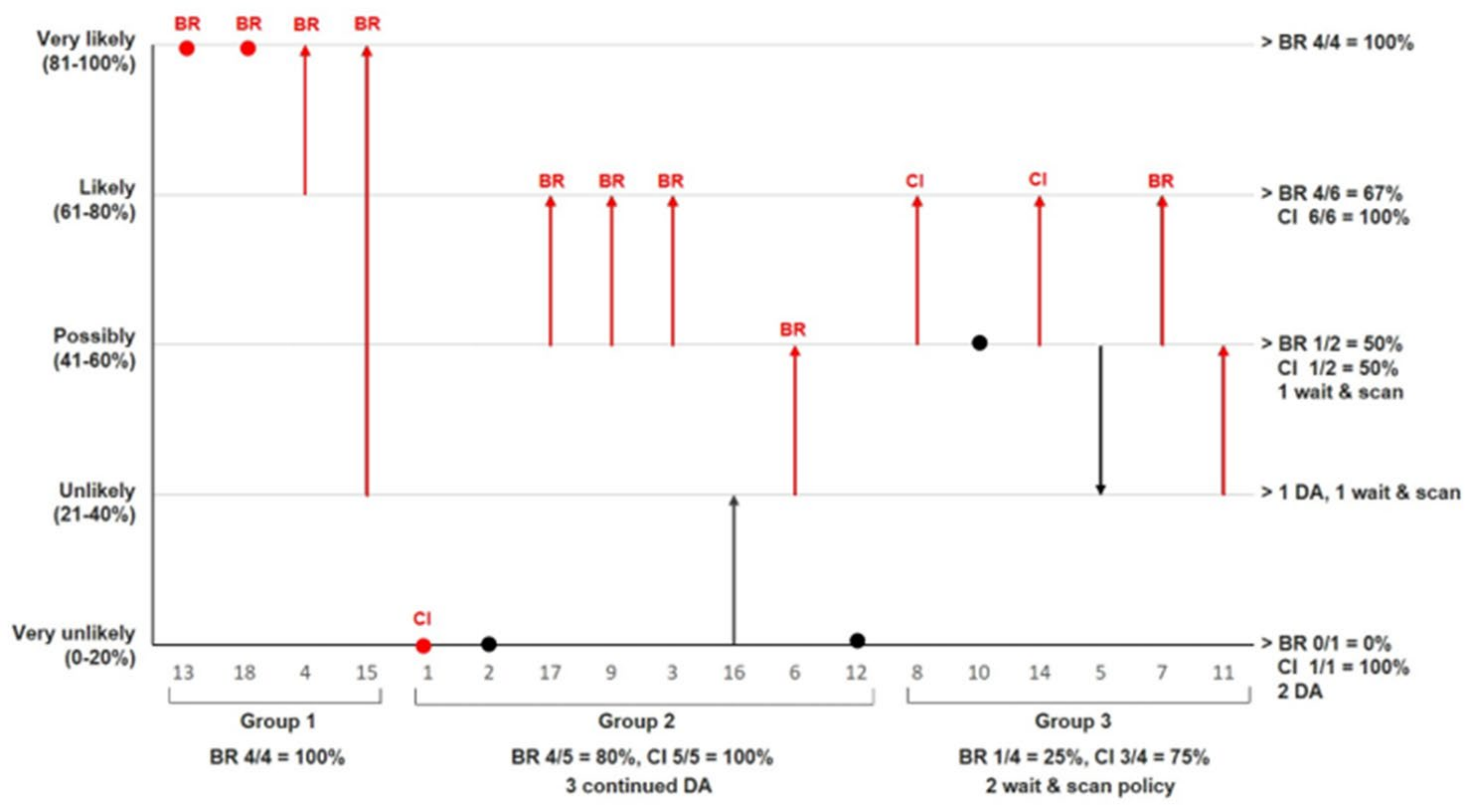
Estimated remission chance before and after Met-PET/MRI^CR^. On the x-axis all patients per group: 1 = Met-PET/MRI^CR^ for confirmation, 2 = MET-PET/MRI^CR^ for additional information, 3 = Met-PET/MRI^CR^ for diagnosis. On the y-axis the estimated remission chance. In red patients who underwent transsphenoidal surgery, in black patients who underwent other treatment. The bottom and head of the arrows show the estimate before and after Met-PET/MRI^CR^, respectively. A dot indicates the estimate did not change. *BR* biochemical remission, *CI* clinical improvement without biochemical remission, *DA* dopamine agonist

Estimated likelihood of remission for the complete group increased significantly after Met-PET/MRI^CR^ as compared to likelihood based on MRI only. The distribution before *vs.* after Met-PET/MRI^CR^ was: very unlikely 22% vs. 17%, unlikely 17% vs. 11%, possibly 44% vs. 17%, likely 6% vs. 33%, very likely 11% vs. 22% (p = 0.005) (Fig. 4). The positive and negative predictive value of a very likely outcome after Met-PET/MRI^CR^ were for: corresponding intraoperative findings 1.0 and 0.0, respectively, positive histology 0.75 and 0.22, respectively, biochemical remission 1.0 and 0.44, respectively, and clinical improvement 1.0 and 0.89, respectively.

**Fig. 5:**
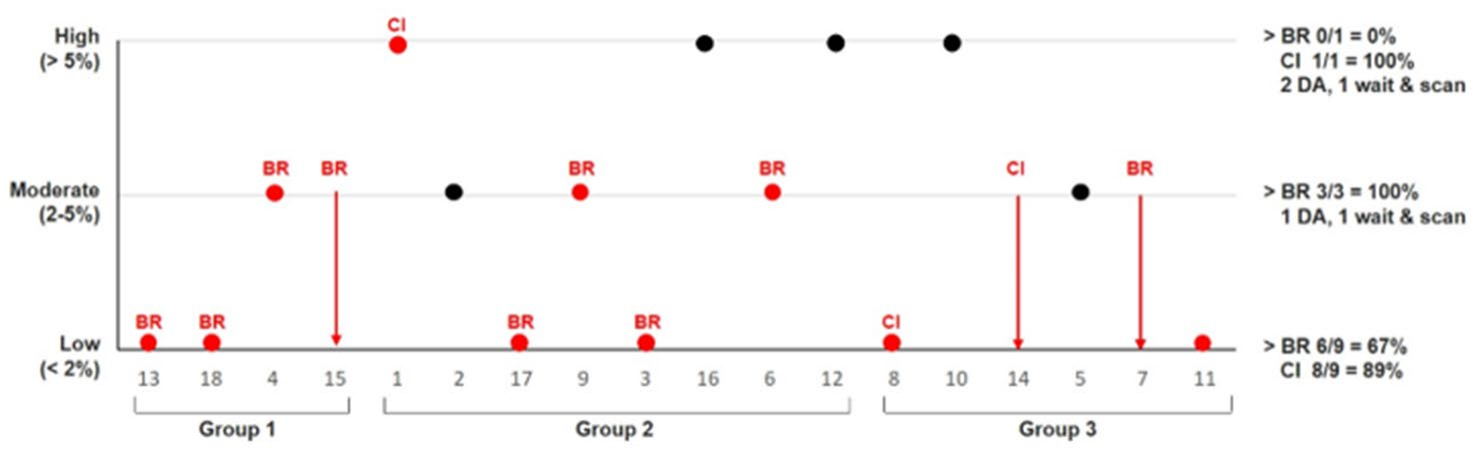
Estimated complication risk before and after Met-PET/MRI^CR^. On the x-axis all patients per group: 1 = Met-PET/MRI^CR^ for confirmation, 2 = MET-PET/MRI^CR^ for additional information, 3 = Met-PET/MRI^CR^ for diagnosis. On the y-axis the estimated complication risk. In red patients who underwent transsphenoidal surgery, in black patients who underwent other treatment. The bottom and head of the arrows show the estimate before and after Met-PET/MRI^CR^, respectively. A dot indicates the estimate did not change. *BR* biochemical remission, *CI* clinical improvement without biochemical remission, *DA* dopamine agonist

Estimated complication risk for the complete group did not change significantly following Met-PET/MRI^CR^ (Fig. 5). The distribution before *vs.* after Met-PET/MRI^CR^ was: low 33% vs. 50%, moderate 44% vs. 28%, high 22% vs. 22% (p = 0.083). Complication rate was low: 6/13 patients (46%) had transient complications (i.e. mild transient DI or SIADH) [group 1: 1/4 (estimated moderate risk), group 2: 2/5 (both estimated moderate risk), group 3: 3/4 (all estimated low risk)], and only 1/13 patients (7%) (subject 6, group 2 (1/5), complex surgery with estimated moderate risk) had long-term complications (partial adrenal insufficiency, unexplained persistent postoperative headache (uncertain relation to TSS)). Looking per risk group, all nine patients in the estimated low risk group were operated, of which three had a transient complication (33%). In the estimated moderate risk group three of five patients (60%) were operated, of which three had a transient complication (100%) and one (subject 6) had long-term complications (33%, 7% of total operated group). In the estimated high-risk group only one of four patients (25%; subject 1, debulking surgery) was operated with an uncomplicated course.

## Discussion

This paper concludes that combined structural and functional imaging with a more accurate visualization of the size and extension of the adenoma and iterative MDT discussions provides added value in selected cases for prediction of surgical success and so clinical decision making. For patients with prolactinoma with drug intolerance or resistance, surgery is the next step in the treatment algorithm. Based on recent evidence, surgery is now increasingly considered as a first line treatment option for microprolactinomas and circumscribed macroprolactinomas in which complete resection can be anticipated [2, 5, 14–30]. With more elective cases being considered for surgery, it becomes more clinically relevant to reliably identify the precise localization of the (remnant) lesion, possible multifocality, and its potential invasion in surrounding structures. Optimal imaging can improve the estimation of the likelihood of successful (revision) surgery or complications.

The potential utility of Met-PET(/MRI^CR^) in guiding management in pituitary adenomas has previously been reported by several groups [6–9, 31–37]. The ability of MET-PET(/MRI^CR^) to localize sites with abnormal tracer uptake varies from 67 to 100%, depending on the type of pituitary adenoma and setting. Recent studies from the Cambridge group correlating imaging findings with subsequent treatment decisions and clinical outcomes in patients with specific functional pituitary adenomas (Cushing’s disease and acromegaly) have shown clear added value of Met-PET/MRI^CR^ for treatment decision making [6–9]. Up to now no similar studies have been performed in patients with prolactinoma with current methodology with MRI co-registration, however, some earlier series using Met-PET included prolactinoma cases, demonstrated applicability in this tumour type as well [31, 33, 35–37]. Bergström et al. showed rapid decrease in amino acid metabolism on Met-PET after starting dopamine agonist treatment, which was later accompanied by tumour shrinkage [37]. In 1991 they reported their experiences in over 400 PET studies in patients with pituitary adenomas, concluding that Met-PET can provide valuable complementary information in the diagnosis of the adenoma [36]. The highest tumour-to-brain ratio of methionine uptake was found in prolactinomas, increasing linearly with serum prolactin level, reaching values up to 9.0 in very active prolactinomas [36]. Similar findings were reported by Muhr in 2006 who described the use of Met-PET in 165 patients with pituitary adenomas, of which 30% were prolactinomas [33]. In a study of Tang et al. Met-PET was positive in 8 out of 10 patients with recurrent or persistent prolactinoma; the two patients with negative Met-PET were scanned under uninterrupted DA treatment. Again, a strong correlation was found between the activity volume index and the serum prolactin level [31]. Finally, in a retrospective study by Feng et al. in 2016, Met-PET was performed in 10 patients with prolactinoma followed by TSS, and found a 100% sensitivity and 95% specificity of Met-PET for detection of prolactinoma [35].

In the current study we report the learning curve and ‘real life’ experience of our Pituitary MDT with this technique that we adapted in 2018 in collaboration with Cambridge. The MDT decided to perform Met-PET/MRI^CR^ only when we felt that the extra information would have potential therapeutic consequences. Consequently, this is not a consecutive case series of prolactinoma patients seen at our centre, nor a consecutive series of prolactinoma cases counselled for surgery. It is a consecutive series of selected complex cases, with difficult remnants and difficult to delineate prolactinoma. Therefore, the take home message is not our remission rate or complication rate, but the importance of presurgical planning and the development of our clinical reasoning and learning curve since we believe this is essential in this process. We aimed to report our experience, caveats, and successes of incorporation of this new diagnostic modality in the treatment algorithm of complex cases referred to our Pituitary Centre. In general, we consider the results achieved in this difficult patient group as a very high remission rate when taking in to account the complexity of the cases, e.g. reoperation, localization near cavernous sinus, inconclusive MRI for adenoma.

Based on our findings in this study we conclude that:

- Met-PET/MRI^CR^ is highly sensitive for detection of virtually all (non-cystic) prolactinomas, with clear difference from MR imaging (completely corresponding in 28%, partially corresponding in 50%, and non-corresponding in 22% patients). After reassessment, five Met-PET/MRI^CR^ identified lesion(s) could retrospectively also be identified on MRI. Operative findings were compatible with Met-PET/MRI^CR^ in 92%. In this complex case series, all but one patient had a positive outcome as defined by normalization of prolactin level and/or clinical improvement, with a low long-term complication rate.
- Assessment of ^11^C-methionine uptake is a useful tool in complex prolactinomas cases because uptake is higher than in normal pituitary tissue in most cases. A single case without increased uptake had less clear findings during surgery and postoperatively.
- Met-PET/MRI^CR^ in prolactinoma provides additional information on the anatomical relationship of the tumour with the cavernous sinus, which can help inform the likelihood of complete surgical resection.
- Met-PET/MRI^CR^ provides reliable information in the following situations: confirmation of functionality of small intrasellar recurrent adenoma also seen on MRI, and confirmation and exclusion of multifocality in cases with several possible small remnants of a previous macroadenoma in an enlarged, partly empty sella.
- Met-PET/MRI^CR^ is associated with additional costs, not only with respect to performing the scan, but also in terms of the time and expertise required to critically interpret the result. Therefore, the indication for this additional scan after protocolled MRI should be thoroughly discussed in the MDT before being requested.
- It is important to distinguish the different subgroups of patients preoperatively because they have different challenges.
- The multidisciplinary discussion for complex cases associated with the implementation of this new technique is another factor that may be associated with the high remission rates.

It is important to acknowledge that there are several potential situations in which functional imaging may fail to identify and localize a (residual) adenoma with Met-PET/MRI^CR^. These include (i) a cystic lesion (as observed in our series, case 13), (ii) when serum prolactin is low (for example when there is suppressed tumour activity due to continued DA use/too short withdrawal period/little active prolactinoma), (iii) technical issues (low tracer activity, co-registration mismatch), (iv) hyperplasia instead of adenoma. In contrast to somatostatin analogue withdrawal in acromegaly patients [8], currently there is no agreed consensus for how long in advance DA should be suspended prior to Met-PET/MRI^CR^, but based on the half-life of cabergoline, the dose, and its biological effects, a period of 4 weeks is recommended, which can be extended if there is insufficient elevation of prolactin. In our cohort, prolactin levels at the time of Met-PET/MRI^CR^ varied between 1.4 and 161 times ULN. In our series, all patients with mildly elevated prolactin levels (between 1.4 and 2.5 times ULN) had positive Met-PET findings and achieved biochemical remission after surgery, suggesting that even in prolactinoma cases with mild prolactin elevation Met-PET can be diagnostic. Finally, availability and quality of pituitary MRI at diagnosis (i.e. pre-treatment) may be essential for optimal interpretation of Met-PET/MRI^CR^.

Based on our experiences and this evaluation we suggest the following guidance for Met-PET/MRI^CR^ indications for the particular clinical situations in our centre as subdivided in our paper:

Surgery naive patients referred for surgery with an obvious and well-defined adenoma on MRI have a high pre-test likelihood of remission and low risk of complications, and therefore we recommend against routine Met-PET/MRI^CR^ (*group 1*), but in individual cases it may be requested when there is a particular high need to weigh surgical chances against alternatives. In patients with clear intrasellar remnants following previous surgery (*group 1*), Met-PET/MRI^CR^ was helpful in confirmation, however, in time, with the obtained increase in our learning curve, we feel that an increasing subset of this group may also be operated without Met-PET/MRI^CR^ guidance.

We would certainly recommend Met-PET/MRI^CR^ in cases with close relation or potential CSI, to broaden the preoperative scope on surgical perspective for a more accurate assessment of the benefit-risk ratio of TSS. This would include both for counselling on first surgeries and reoperations. The advice obtained after Met-PET/MRI^CR^ can be used to balance surgical need, against chances for remission (*group 2*).

For patients with a difficult remnant after surgery or tumour shrinkage after long-term medical therapy (*group 2*), we recommend functional imaging to distinguish suspected remnant from post-treatment changes (e.g. sella remodelling due to scar tissue) and to screen for multifocality. This is clearly the most difficult group, but with potentially the greatest added value of Met-PET/MRI^CR^. For patients with complex macro-giant adenomas with a high need for treatment because of DA intolerance and/or resistance with a low a priori chance of remission, we have used Met-PET/MRI^CR^ in order to delineate the most active sites of residual adenoma that are potentially amenable to Met-PET-guided debulking surgery or targeted radiotherapy (*group 2*). We deemed this strategy helpful to counsel and plan the surgery, however, still, these surgeries appear to be highly difficult and visualization is limited by different layers, lamelles and/or pockets. Therefore, the intraoperative judgment is impaired and although Met-PET/MRI^CR^ may guide the plan, the extent of resection is dependent on the situation encountered during surgery. We will proceed with Met-PET/MRI^CR^ evaluations in those cases to collect more data to correlate with postoperative findings. Consequently, at present we cannot draw meaningful conclusions on the potential added value of Met-PET/MRI^CR^ in this group.

Finally, in *group 3*, Met-PET/MRI^CR^ was helpful in the interpretation of negative/equivocal MRI (i.e. when the remnant adenoma was not readily visualized). This is considered a difficult group, with a potentially different type of prolactinoma, requiring further study as to whether these cases are candidates for surgery or not due to multifocal disease or hyperplasia rather than adenoma. Our results warrant further analysis and until then we will continue to perform scans but will be restrictive with proceeding for surgery—only in case of a very high need—since the outcomes of this particular group are less certain than for clearly visible lesions.

This study has several limitations. The sample size is small, and the clinical indications vary. Larger cohorts, from several centres in different countries/continents are required to further delineate the role of Met-PET/MRI^CR^ in the management of prolactinoma in particular for the indications specified above. An important drawback of Met-PET/MRI^CR^ is the short half-life of ^11^C-methionine (approx. 20 min), which means that the scan can only be acquired in centres with an adjacent cyclotron. Use of alternative tracers might be a solution. Radiolabelled amino acid ligands such as ^18^F-Fluoro-Ethyl-Tyrosine (^18^F-FET) have not yet proven efficacy in pituitary adenomas but exhibit a comparable uptake mechanism and pattern in brain tumours and have logistic benefit over ^11^C-methionine because of the longer half-life of Fluor-18 enabling transportation to centres without a cyclotron. Any functional imaging requires the MDT setting for dedicated evaluation of added value.

**Fig. 6:**
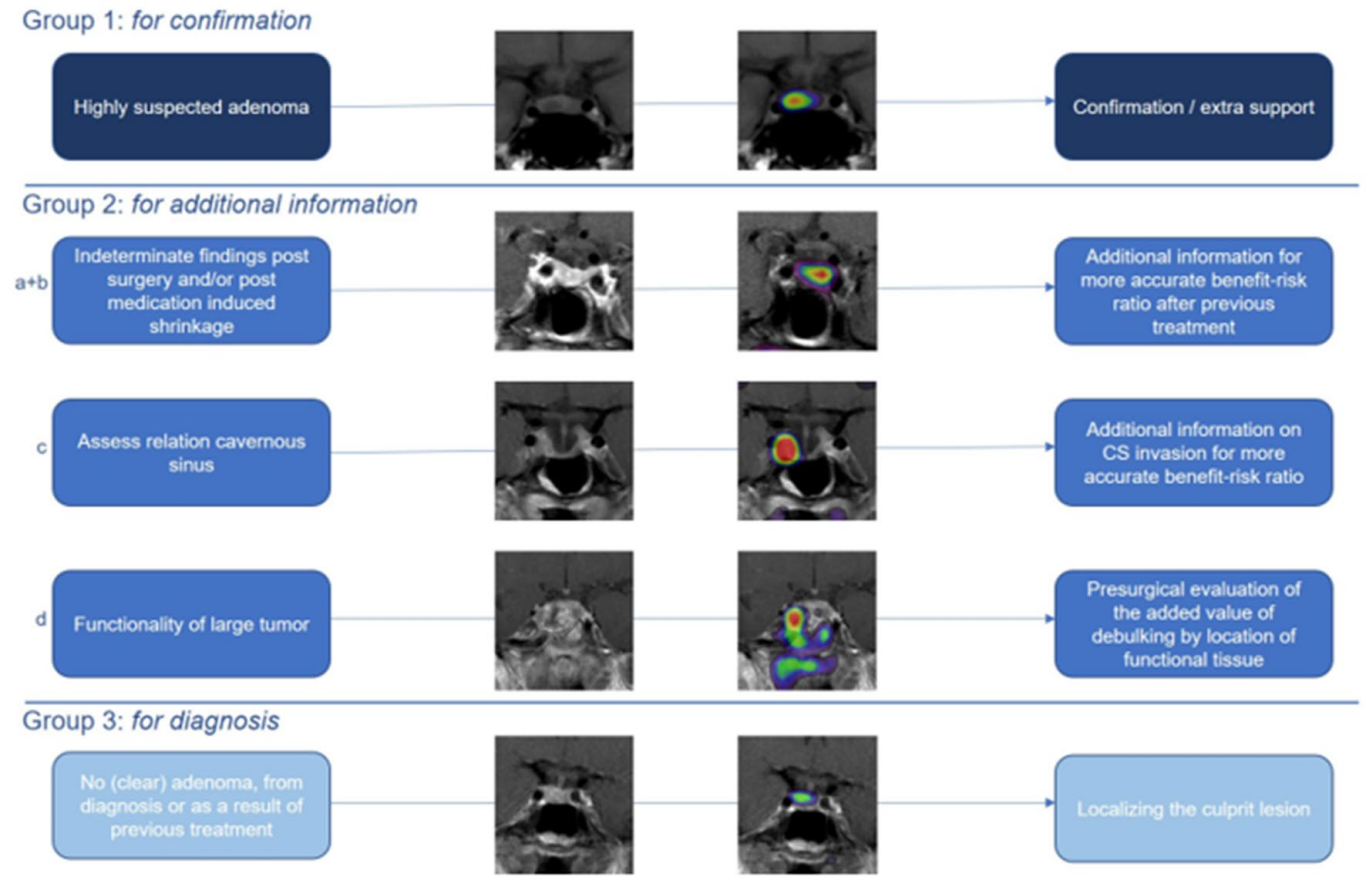
Situations in which Met-PET/MRI^CR^ may help inform decision-making in patients with prolactinoma with dopamine agonist intolerance and/or resistance and/or preference for surgery. Group: 1 = Met-PET/MRI^CR^ for confirmation, 2 = MET-PET/MRI^CR^ for additional information, 3 = Met-PET/MRI^CR^ for diagnosis

In summary (see Fig. 6): Met-PET/MRI^CR^ is highly sensitive in (1) all patients with a well-defined non-cystic prolactinoma (remnant) and so this group is a suitable group to “test” and gain experience with functional imaging and the multidisciplinary collaboration. Met-PET/MRI^CR^ can provide clear added value in the clinical decision-making process for prolactinoma considered for surgery to assess chances and risks in: (2a) with recurrent or persistent disease after surgery and an adenoma suspected on MRI (to discriminate postoperative remodelling from a remnant), (2b) remnant prolactinoma tissue after drug induced volume decrease (to confirm location of remnants in enlarged sella and exclude other spots), and (2c) prolactinoma with uncertain extension/invasion to the cavernous sinus (more accurate benefit-risk ratio of TSS). Met-PET/MRI^CR^ can provide potential added value in: (2d) patients with (previous) large and giant prolactinoma without certain invasion (to delineate the most active sites of residual adenoma that are potentially amenable to Met-PET-guided debulking surgery or targeted radiotherapy), (3) patients without clear adenoma, or only somewhat asymmetrical enlarged pituitary (similar to Cushing disease cases without clear adenoma). Both indications require further evaluation and at present therefore should only be considered when there is a high need for surgery.

## Supplementary Information

Below is the link to the electronic supplementary material.

Supplementary file1 (PDF 192 KB)

Supplementary file2 (PDF 205 KB)

Supplementary file3 (PDF 696 KB)

## Data Availability

The datasets generated during and/or analysed during the current study are available from the corresponding author on reasonable request.
