# Supplementary material for: Implementation of functional imaging using 11C-methionine PET-CT co-registered with MRI for advanced surgical planning and decision making in prolactinoma surgery
Source: Pituitary. 2022 May 26;25(4):587–601. doi: 10.1007/s11102-022-01230-2 (PMC9345807; doi:10.1007/s11102-022-01230-2)
Supplement: Supplementary file 1 — Supplementary file1 (PDF 192 KB) [file 11102_2022_1230_MOESM1_ESM.pdf]

Online Resource 1: Case descriptions

Implementation of functional imaging using ^{11}C-methionine PET-CT co-registered with MRI for advanced surgical planning and decision making in prolactinoma surgery

Pituitary

Leontine E.H. Bakker*, Marco J.T. Verstegen*, Idris Ghariq, Berit M. Verbist, Pieter J. Schutte, Waiel A. Bashari, Mark C. Kruit, Alberto M. Pereira, Mark Gurnell, Nienke R. Biermasz, Wouter R. van Furth‡, Lenka M. Pereira Arias-Bouda‡

Corresponding author: Leontine E.H. Bakker, Leiden University Medical Center, Leiden, The Netherlands, Department of Medicine, Division of Endocrinology and Center for Endocrine Tumors Leiden (CETL), Pituitary Center, l.e.h.bakker@lumc.nl

GROUP 1: Met-PET/MRI^{CR} for confirmation (subjects 4, 15, 13 and 18) (Online Resource 3 Supplementary Fig. 1)

Subject 4:

Female patient in her fifties was diagnosed with a macroprolactinoma in the nineties and was initially treated with dopamine agonists (DA). Because of intolerable side effects transsphenoidal surgery (TSS) was performed two years after diagnosis. Several years later hyperprolactinemia recurred and DA therapy was resumed. Multiple withdrawal attempts in the following years were unsuccessful while experiencing unacceptable side effects. Her prolactin levels were only mildly increased (3.0 times upper limit of normal (ULN)). MRI showed either unchanged postoperative changes or remnant adenoma on the right side (no cavernous sinus invasion (CSI)). To confirm localization of the remnant, Met-PET/MRI^{CR} was performed which showed evidence for a residue anterolaterally in the sella on the right side in close proximity of the cavernous sinus. Her estimated chance of remission with surgery was adjusted from likely to very likely. Surgical and histopathological findings confirmed a lactotroph adenoma, and prolactin normalized within one week. Given a revision TSS and long history of medical treatment, Met-PET/MRI^{CR} was of added value to gain more certainty.

Subject 13:

Female patient in her late teens presented at our center with secondary amenorrhea and was diagnosed with a cystic macroprolactinoma (prolactin 4.9x ULN). She was treated with DA for only 2 months, which she had to discontinue because of an allergic reaction to both cabergoline and quinagolide. Conventional imaging showed a clearly delineated right-sided intrasellar cystic adenoma of 12 mm with CSI (Knosp 2). Met-PET/MRI^{CR} however showed no concordant uptake of methionine. TSS was performed with corresponding intraoperative findings. Histopathological findings were inconclusive with predominant staining for prolactin and growth hormone but without clear proof of adenoma (probably due to the cystic lesion), but serum prolactin levels normalized within one week. In a well-defined cystic functional adenoma the added value of Met-PET/MRI^{CR} is probably limited by the fact that there is virtually no uptake of methionine in cystic lesions. Of note, given the moderately increased prolactin level at diagnosis and the negative scintigraphic and histopathological findings, a cystic nonfunctional macroadenoma or a cyst with stalk compression, instead of a macroprolactinoma, cannot completely ruled out. However, the fact that her menstrual cycle normalized postoperatively and that she experiences clear clinical improvement argue more for a prolactinoma.

Subject 15:

Male patient in his thirties presented with gynecomastia and was diagnosed with a microprolactinoma (prolactin 6x ULN). He was started on cabergoline, but was referred to our center three years later because of a preference for surgery. However, despite a clear lesion on the left side of the sella on MRI, TSS was unsuccessful with negative histopathology and unchanged prolactin level. Given this unexpected outcome, the likelihood of remission with revision TSS was estimated as unlikely, and functional imaging was used to discriminate between residual adenoma and postoperative remodeling. Met-PET/MRI^{CR} showed clear tracer uptake on the left side, increasing estimated remission chance to very likely. He underwent revision TSS with perioperative findings concordant with imaging and positive histopathology. Prolactin normalized within one week. No complications occurred.

Subject 18:

Female patient in her twenties was diagnosed with a macroprolactinoma and was initially treated with a DA for a total duration of 52 months. She was referred to our center because of significant side effects of the medication. Conventional imaging showed a well-defined right-sided intrasellar adenoma of 10 mm without CSI. Met-PET/MRI^{CR} was completely concordant with MRI. She underwent TSS with intraoperative findings concordant to imaging. Histopathological findings showed typical adenoma, and immunohistochemical staining was positive for prolactin. Accordingly, serum prolactin levels normalized within one week. No complications occurred, and menstrual cycle restored within 6 weeks. In similar future cases with a clear adenoma on MRI with a high likelihood for cure, low anticipated risk for complications and a high need for surgery, we will refrain from functional imaging.

GROUP 2: Met-PET/MRI^{CR} for additional information (subjects 1, 2, 3, 6, 9, 12, 16 and 17) (Online Resource 3 Supplementary Fig. 2)

Subject 1:

Female patient in her twenties was diagnosed with an intrasellar macroprolactinoma with CSI (at least Knosp 3B). Treatment with cabergoline was initiated resulting in a partial biochemical response but no convincing radiological response, and at the expense of side effects. Consequently, she was referred to our center. Conventional imaging was of moderate quality and showed an intrasellar tumor remnant of 24 mm with bilateral invasion in the cavernous sinus (Knosp 3B) right more than left, however lateral extension of the remnant was difficult to assess. The likelihood of remission with surgery was estimated to be very unlikely, with a high risk of complications. For a better assessment of the extension in both cavernous sinus, and hence, the likelihood, if any, of remission after TSS, we decided to perform Met-PET/MRI^{CR}. On Met-PET/MRI^{CR} methionine uptake was seen in the right cavernous sinus, but not in the left. We discussed her in the Cambridge-Leiden MDT. Treatment proposal was surgery with a high chance of residual small remnant in the right cavernous sinus (estimated chance of remission very unlikely, complication risk moderate) which most likely could be controlled with low dose cabergoline treatment, or by stereotactic radiotherapy. Subsequent surgery had concordant perioperative findings, histopathological confirmation, and maximal surgical result. As expected, normoprolactinemia was not reached, but she experienced significant clinical improvement, and on low dose cabergoline treatment she recently became pregnant (preoperative prolactin 54.9x ULN, postoperative 14.5x ULN without DA and 2.5x ULN after starting cabergoline 0.25 mg two times a week with restoration of menstrual cycle and shortly after pregnant). The added value of Met-PET/MRI^{CR} in this specific case was evident, as it improved estimations of inability to achieve remission, and hence counseling to the patient.

Subject 2:

Male patient in his twenties was diagnosed with a macroprolactinoma with supra- and parasellar extension (max diameter 37 mm with bilateral CSI (probably Knosp 4, however poor quality of initial scan) and visual field defects). He was started on cabergoline with biochemical and radiological response, but at the expense of significant side effects (pathological gambling urge) Therefore, he was operated at our center (positive histopathology) with the objective to achieve, though unlikely, remission, or at least significant debulking in an attempt to significantly reduce drug need. As anticipated, postoperative prolactin level decreased but did not normalize, with accompanying symptoms. Early postoperative MRI was difficult to interpret due to postoperative changes but was suspected for a possible remnant in the left cavernous sinus (possible Knosp 3B?). The likelihood of remission with revision TSS was estimated to be very unlikely with a moderate risk of complications. Given the severe side effects of DA, his still intact pituitary function and the uncertainty of localization

and extension of the remnant adenoma, Met-PET/MRI^{CR} was used to optimize treatment decisions (surgical options? radiotherapy?). Met-PET/MRI^{CR} showed increased uptake left anteriorly in the sella and slightly increased uptake in both cavernous sinus (right more than left), suspected for residual adenoma. We discussed him in the Cambridge-Leiden multidisciplinary team (MDT). The likelihood of remission and complication risk did not change. It was concluded that revision TSS was not a feasible option because of no added value. A test of cabergoline treatment was restarted at the lowest possible dose (0.25 mg twice a week (vs. max 150 mcg quinagolide daily before operation), which resulted in both biochemical (near normal prolactin: 1.5x ULN) and radiological regression, and is now well-tolerated without relevant side effects (current dose 0.25 mg four times a week). In case of escape of medical therapy and/or intolerance in the future, radiotherapy will most probably be the next step. We considered Met-PET/MRI^{CR} of added value in this case in optimizing treatment decisions and patient counseling.

Subject 3:

Female patient in her thirties was diagnosed with microprolactinoma and was treated with DA of which she experienced numerous side effects. Multiple attempts to withdraw DA were unsuccessful. Ten years after diagnosis she was referred to our center because of a wish for surgical treatment. No initial pre-treatment imaging was available. MRI was uncertain, but suspected for a residual adenoma on the right side (Knosp 0), and a possible second small lesion on the sellar floor on the left side (residual adenoma or partial volume effect?). Met-PET/MRI^{CR} was performed to confirm functionality of the lesion on the right side and to assess multifocality. There was clear increased uptake in the lesion to the right without any other suspected uptake. We discussed her in the Cambridge-Leiden MDT. Estimated likelihood of remission increased from possibly to likely, and estimated risk of complication remained low. Surgical and histopathological findings confirmed a lactotroph adenoma, and prolactin levels normalized within one week. No complications occurred. Met-PET/MRI^{CR} was of added value in this patient with longstanding medical treatment, unavailable pretreatment MRI and indeterminate MRI findings, as it ruled out multifocality

Subject 6:

Male patient in his fifties was diagnosed with a macroprolactinoma in the late nineties and had been treated with DA since then, but with persistent hyperprolactinemia and accompanying symptoms and with significant side effects. No initial MRI was available. Based on his latest MRI, showing clival invasion and CSI Knosp 3b on the left, plus his longstanding medical treatment, likelihood of remission with TSS was estimated to be unlikely with a moderate estimated risk of complications. Met-PET/MRI^{CR} was used to assess the extension of the remnant adenoma and the most active part as a target for further therapy, showing tracer uptake left dorsocaudally with CSI, and changing his estimated chance of remission to possibly. We discussed him in the Cambridge-Leiden MDT. Given his intolerance and resistance to DA, it was deemed reasonable to undertake surgical exploration. During surgery highly fibrous tissue was found and the dura was infiltrated with tumorous tissue at multiple sites; dura possibly infiltrated with adenoma was left behind. The pituitary was identified on the right side. Histopathological findings confirmed a lactotroph adenoma. Quite unexpectedly, he achieved a normal prolactin level. Postoperative course was complicated by a transient n.VI paralysis and diabetes insipidus (DI), and hitherto partial adrenal insufficiency and unexplained persistent headache (uncertain relation to TSS).

Subject 9:

Female patient in her twenties was diagnosed with a macroprolactinoma (no available scan at diagnosis). She was operated elsewhere within one year after diagnosis without any information on

histology and outcome. Three years later she was operated again (positive histopathology) after which she reached biochemical remission. In between she was treated with cabergoline but experienced significant side effects. Again three years later she was referred to our center because of suspected recurrence. MRI possibly showed a small remnant on the left side (Knosp 2), and possibly also on the right side. Estimated chance of remission with revision TSS was possibly with a moderate estimated risk of complications. Met-PET/MRI^{CR} was used to localize remnant adenoma and assess the right sided extension. It showed clear increased uptake in the sella on the left side. Chance of remission estimate changed to likely, risk of complication remained moderate. She underwent revision TSS with histopathological confirmation of adenoma, and positive immunohistochemical staining for both prolactin and growth hormone (GH), after which her prolactin level completely normalized. Apart from transient DI the postoperative course was uncomplicated. In this case Met-PET/MRI^{CR} was of added value in helping to distinguish between residual functional adenoma and post-treatment remodeling.

Subject 12:

Male patient in his late teens was diagnosed with a macroprolactinoma (prolactin 100x ULN) and was started on cabergoline. Because of regrowth while on DA despite previous shrinkage, debulking surgery was performed followed by a fast revision operation because of apoplexia in the remnant with n. VI paralysis on the right side, which resolved postoperatively. Based on the course and a new MRI, likelihood of remission with surgery was estimated as very unlikely (CSI right Knosp 4) with a high estimated risk of complications. Because of his young age, drug side effects and suspicion of an aggressive tumor, functional imaging was used to assess the possibilities of a third, curative surgery, or if that would be deemed impossible to assess the most active part as target for further therapy (debulking surgery, radiotherapy). Met-PET/MRI^{CR} showed several sites with increased tracer uptake including CSI laterally of the internal carotid artery. Hence, revision surgery was not an option. Currently we re-initiated medical therapy in an attempt to postpone the necessity for radiotherapy.

Subject 16:

Male patient in his late fifties was diagnosed with a macroprolactinoma in the eighties and had been treated with DA for a very long time, of which he was experiencing significant side effects negatively affecting quality of life (QoL). Given uncertain findings on MRI, Met-PET/MRI^{CR} was performed to explore surgical options. Increased methionine uptake was seen left dorsolaterally with possible CSI, and a suspected lesion right dorsolaterally without CSI. Remission chances with TSS were estimated to be unlikely with a high complication risk. However, given his high burden of disease after longstanding medical treatment TSS was considered a realistic option for improving QoL. Nevertheless, the patient decided to continue with DA for now.

Subject 17:

Female patient in her thirties underwent emergency TSS elsewhere for possible apoplexy in a newly diagnosed macroprolactinoma after which she was started on cabergoline. A baseline MRI was not available. Nine years later she was referred to our center to assess surgical options, because of persisting side effects of medical therapy. Conventional MRI showed suspected right-sided intrasellar remnant of 11 mm (probable Knosp 1), on the left side it was not clear whether normal pituitary tissue, post-treatment remodeling, or remnant was present. The likelihood of remission after revision TSS was estimated to be possible with a low complication risk. To gain more insight in CSI and the possibility of multifocality Met-PET/MRI^{CR} was performed, which showed methionine uptake only on the right side in the sella, next to the cavernous sinus without invasion, suspected for remnant adenoma. Likelihood of remission estimate increased to likely, with no change in estimated risk of complications (low). She was re-operated, and histopathology confirmed prolactinoma, and prolactin normalized within one

week postoperatively. No complications occurred. Met-PET/MRI^{CR} evidently improved preoperative assessment on surgical perspective.

GROUP 3: Met-PET/MRI^{CR} for diagnosis (subjects 5, 7, 8, 10, 11 and 14) (Online Resource 3 Supplementary Fig. 3)

Subject 5:

Female patient in her thirties was diagnosed with a microprolactinoma (prolactin 4.0x ULN) in a workup for subfertility. She was treated with DA. Because of significant side effects she stopped the medication five years later with recurrence of the hyperprolactinemia. Because of equivocal findings on MRI (lesion on right and left side?), a 7T-MRI-scan was performed, which confirmed a lesion on the left side with a linear structure reaching to midline. She was operated five years after diagnosis with positive histopathology but no normalization of her prolactin level. Postoperative MRI was inconclusive with suspicion of a possible small remnant on the right side. Met-PET/MRI^{CR} showed tracer uptake left anterolaterally with evident CSI (between legs internal carotid artery), decreasing the likelihood of remission from possibly to unlikely. She was discussed in the Cambridge-Leiden MDT and a wait-and-scan policy without DA treatment was proposed, and considered to be the current best management for her (given the outcome of prior surgery, localization of remnant, and expectation management in a vulnerable patient). If disease progresses DA or radiotherapy are probably preferred over surgery, however current imaging will guide final treatment decision.

Subject 7:

Female patient in her thirties presented with secondary amenorrhea and galactorrhea and was diagnosed with a prolactinoma (prolactin 6x ULN). She was treated with cabergoline with however significant side effects. MRI did not show a clearly visible lesion (right, left?). Met-PET/MRI^{CR} showed increased tracer uptake on the right side without any uptake elsewhere. Cabergoline was stopped at least three weeks prior to the scan and her prolactin level was 2.8x ULN at the time of Met-PET/MRI^{CR}. Remission chances were considered to be likely with low complication risk. She underwent TSS. Intraoperatively, surgical vision was suboptimal due to bleeding from the cavernous sinus, with a probable identification of adenoma tissue perioperatively on the right side. Histopathology confirmed an adenoma with strongly positive immunohistochemical staining for GH and less for prolactin. Subtle clinical improvement was achieved, and prolactin level did drop from 6x ULN preoperatively to ~2x ULN postoperatively. At that moment, in the early days of Met-PET/MRI^{CR} availability in our center, we had some doubt about the sensitivity of the scan due to the possibility of insufficient withdrawal of cabergoline, and decided to revision surgery aimed at removing a suspected (double or remnant) adenoma on the left side identified on conventional MRI but not positive on the functional imaging. This was an exploration while showing identical histopathology, without any impact on the prolactin level nor the complaints. There was a critical re-appraisal of this case in the Leiden/Cambridge MDT and we concluded that the remnant lesion was visible at the right cavernous sinus level. This was confirmed with a second Met-PET/MRI^{CR} (prolactin 2.4 times ULN) and this lesion highly suspicious for a remnant on the right side dorsolaterally (axial view), with a likely estimated remission chance and a low estimated complication risk, was surgically resected successfully with a low postoperative prolactin level and uneventful course.

Subject 8:

Postmenopausal female patient in her fifties was diagnosed with a prolactinoma. She was treated with cabergoline for several years, but experienced side effects. MRI-scan was inconclusive, and no initial MRI-scan was available. Met-PET/MRI^{CR} showed tracer uptake on the left side without CSI, increasing the likelihood of remission from possibly to likely with an estimated low risk. Surgery was performed with positive histopathology. Her prolactin level decreased but did not normalize (preoperatively 6.8x ULN vs. 2.6x ULN postoperatively (both without DA)), however she did have significant clinical improvement. Besides transient DI no complications occurred.

Subject 10:

Female patient in her twenties was diagnosed with a macroprolactinoma (prolactin 48x ULN) and was initially treated with DA. Because of intolerance she stopped medication and was operated in our center six years after diagnosis with positive histopathology and normalization of her prolactin level. However, after one year her prolactin level started to increase slowly. MRI was inconclusive with suspicion of a possible remnant on the left side. Met-PET/MRI^{CR} (prolactin 5.5x ULN) showed uptake left dorsocaudally, but also right ventrocranially, without CSI. Retrospectively these sites were also identified on previous MRI. Likelihood of remission was estimated as possibly given multifocality and prior TSS, with a high estimated risk for complications due to previous surgery and DA treatment and localization of the pituitary gland. However, given her intolerance and resistance for DA revision TSS was advised. Nevertheless, for now, the patient preferred a wait and scan policy without DA for now.

Subject 11:

Female patient in her thirties presented with secondary amenorrhea and hyperprolactinemia (prolactin 19x ULN) and was diagnosed with a microprolactinoma. She was started with DA therapy of which she experienced significant side effects. Switch to other dopamine agonists and stop attempts were unsuccessful. Baseline MRI was of very poor quality, and a current MRI did not show a visible remnant. Hence, functional imaging was performed (prolactin 3.9x ULN). Scintigraphic findings were equivocal with asymmetrical uptake in the sella left more than right and stalk deviation to the right, suggesting a remnant on the left side, without CSI. Likelihood of remission was considered as possibly with a moderate chance of complications. She underwent surgery during which an apparently easy to remove firm adenoma on the sellar floor was completely resected, however histopathology was non-diagnostic. Her prolactin level did not decrease significantly (pre- and postoperative prolactin ca. 3.8x ULN, both without DA), although clinically she did experience improvement in her menstrual cycle and mental state. Apart from transient DI no other complications occurred. New imaging (MRI and functional imaging) is suspected for a residual adenoma on the left side. Because of an active pregnancy wish a low dose of cabergoline was recently resumed, after which she became pregnant quickly. After delivery we will re-evaluate whether a re-exploration is still an option.

Subject 14:

Female patient in her thirties was diagnosed with a macroprolactinoma. DA treatment was initiated with biochemical and radiological good response, but also with side effects. Six years later she stopped medication because of intolerance, and was referred to our center. Prolactin level was 4.6 times ULN, and MRI was inconclusive. Met-PET/MRI^{CR}, performed to visualize a remnant lesion, showed tracer uptake in the sella right posterolaterally suspected for residual adenoma without evidence for CSI. Estimated likelihood of remission increased from possibly to likely, and estimated risk of complication decreased from moderate to low. She underwent surgery. Intraoperative findings were concordant to imaging and histopathology confirmed a lactotroph adenoma with possible GH co-secretion. Postoperatively prolactin decreased from 4.6x to 1.3-1.7x ULN. She experienced significant clinical improvement and her menstrual cycle restored. Apart from very mild SIADH, postoperative course was

uneventful. Met-PET/MRI^{CR} helped us visualizing the remnant in a patient with longstanding medical treatment and indeterminate MRI findings.
