# Supplementary material for: Implementation of functional imaging using 11C-methionine PET-CT co-registered with MRI for advanced surgical planning and decision making in prolactinoma surgery
Source: Pituitary. 2022 May 26;25(4):587–601. doi: 10.1007/s11102-022-01230-2 (PMC9345807; doi:10.1007/s11102-022-01230-2)
Supplement: Supplementary file 2 — Supplementary file2 (PDF 205 KB) [file 11102_2022_1230_MOESM2_ESM.pdf]

Online Resource 2: Supplementary Table

Implementation of functional imaging using ^{11}C-methionine PET-CT co-registered with MRI for advanced surgical planning and decision making in prolactinoma surgery

Pituitary

Leontine E.H. Bakker*, Marco J.T. Verstegen*, Idris Ghariq, Berit M. Verbist, Pieter J. Schutte, Waiel A. Bashari, Mark C. Kruit, Alberto M. Pereira, Mark Gurnell, Nienke R. Biermasz, Wouter R. van Furth[‡], Lenka M. Pereira Arias-Bouda[‡]

Corresponding author: Leontine E.H. Bakker, Leiden University Medical Center, Leiden, The Netherlands, Department of Medicine, Division of Endocrinology and Center for Endocrine Tumors Leiden (CETL), Pituitary Center, l.e.h.bakker@lumc.nl

Supplementary Table 1. Characteristics per individual patient.

Group	Subject	Sex/Age (decade)	Duration of disease (months)	Initial PRL (x ULN)	MRI findings at diagnosis	Prior treatment	Pituitary hormone deficiencies	MRI findings after prior treatment, before Met-PET/MRI^{CR}	Indication Met-PET/MRI^{CR}	PRL at Met-PET/MRI^{CR} (x ULN)	Met-PET/MRI^{CR} findings	Estimated remission / risk chance pre Met-PET/MRI^{CR}	Estimated remission / risk chance post Met-PET/MRI^{CR}	MDT Cambridge	Treatment proposed / performed	Perioperative findings corresponding to imaging	Histology	Biochemical remission	Clinical improvement	New pituitary deficits / other complications
1	4	F/ fifties	316	3.0	NA	DA 216 months, surgery	No	GQ, suspected sella right or postop change, 7 mm, no CSI	Confirmation, active?	3.0	Tracer uptake right anterolateral in sella, CSI right	Likely / Moderate	Very likely / Moderate	No	Surgery / Surgery	Yes	Confirmative	Yes	Yes	No, temporary DI
1	13	F/ teens	6	4.9	GQ, 12 mm, cystic lesion, sella right, CSI (Knosp 2 right)	DA, 2 months	No	GQ, cystic lesion, 13 mm sella right, CSI (Knosp 1 right)	Confirmation, learning curve (cystic lesion)	4.6	No tracer uptake in cystic lesion	Very likely / Low	Very likely / Low	No	Surgery / Surgery	Yes	Not confirmative	Yes	Yes	No
1	15	M/ thirties	48	6.0	MQ, 7 mm, sella left, no CSI	DA 39 months, surgery	No	GQ, suspected lesion sella left or postop change? 6 mm, CSI (Knosp 1 left)	Confirmation, resectability	3.9	Intense tracer uptake in lesion left, CSI left	Unlikely / Moderate	Very likely / Low	No	Surgery / Surgery	Yes	Confirmative	Yes	Yes	No
1	18	F/ twenties	57	2.5	MQ, 10 mm, sella right, no CSI	DA, 52 months	No	MQ, lesion sella right, 6 mm, no CSI	Confirmation, learning curve	2.5	Tracer uptake in suspected remnant right side, no CSI	Very likely / Low	Very likely / Low	No	Surgery / Surgery	Yes	Confirmative	Yes	Yes	No
2	1	F/ twenties	18	107	MQ, 28 mm, sella left/right, CSI (Knosp >=3b right)	DA, 18 months	Yes	MQ, equivocal findings, sella left/right, 24 mm, CSI (Knosp >=3b right, Knosp 1 left)	Extension, CSI, resectability	43.8	Tracer uptake intrasellar, CSI right (Knosp >= 3b), not left	Very unlikely / High	Very unlikely / High	Yes	Surgery / Surgery	Yes	Confirmative	No	Yes	No

2	2	M/ twenties	25	208	MQ, 37 mm, intra/suprasellar, bilateral CSI (Knosp 4 both sides)	DA 16 months, surgery	Yes	MQ, 6 weeks postop, possible remnant left CS, CSI (Knosp 3b left)	Localization, extension, CSI, resectability	24.5	Intense tracer uptake left anteriorly, slight uptake CS right, no uptake left CS or sellar floor	Very unlikely / Moderate	Very unlikely / Moderate	Yes	Radio-therapy / Medication	NA	NA	NA	NA	NA
2	3	F/ thirties	116	?	NA	DA 108 monthss	No	MQ, sella right, 5 mm, no CSI; possibly left or partial volume?	Localization, multifocality, extension	1.4	Tracer uptake in suspected remnant right, no uptake elsewhere, no CSI	Possibly / Low	Likely / Low	Yes	Surgery / Surgery	Yes	Confirmative	Yes	Yes	No
2	6	M/ fifties	228	22	NA	DA 242 months	No	GQ, intra/infra-sellar (clival), 17 mm, CSI (Knosp 3b left)	Extension, most active part	17.8	Intense tracer uptake left dorsocaudally, CSI	Unlikely / Moderate	Possibly / Moderate	Yes	Surgery / Surgery	Yes	Confirmative	Yes	Yes	Yes: temporary n.VI paralysis and DI; partial AI, unexplained headache
2	9	F/ twenties	90	7.5	NA	DA 7 months, surgery (2x)	No	MQ, equivocal findings, sella left, 5 mm, possibly right? CSI uncertain	Localization, extension, CSI	1.7	Tracer uptake in suspected remnant left, uncertain CSI	Possibly / Moderate	Likely / Moderate	No	Surgery / Surgery	Yes	Confirmative	Yes	Yes	No, temporary DI
2	12	M/ teens	12	100	GQ, 36 mm, intra/suprasellar, CSI (Knosp 4 right, possible Knosp 1 left)	DA 10 months, surgery (2)	Yes	GQ, sella left/right, 25 mm, CSI (Knosp 4 right)	Most active part	161	Tracer uptake in suspected remnant left dorsolaterally in sella, ventro-caudally to resection cavity, sellar floor and CS right with extension laterally of ICA	Very unlikely / High	Very unlikely / High	No	Medication, in future RTP / Medication	NA	NA	NA	NA	NA
2	16	M/ fifties	408	7.2	NA	DA 403 months	No	PQ, sella left/right, CSI uncertain (Knosp 3b?)	Localization, resectability, most active part	6.8	Tracer uptake left dorsolaterally with possible CSI, second suspected lesion right	Very unlikely / High	Unlikely / High	No	Surgery / Medication	NA	NA	NA	NA	NA

											dorsolaterally without CSI									
2	17	F/ thirties	110	10	NA	DA 102 months, surgery	Yes	GQ, sella right, 10 mm, CSI (Knosp 2); possibly left?	Multifocality, extension, CSI, resectability	9.8	Tracer uptake sella right, no uptake elsewhere, no CSI	Possibly / Low	Likely / Low	No	Surgery / Surgery	Yes	Confirmative	Yes	Yes	No
3	5	F/ thirties	66	4.0	GQ, 5 mm, sella right, no CSI	DA 60 months, surgery	No	GQ, equivocal findings, suspected sella right, 3 mm, no CSI	Localization, extension	2.4	Tracer uptake left anterolaterally with CSI ('between legs ICA') suspected for adenoma; no uptake sellar floor right	Possibly / Moderate	Unlikely / Moderate	Yes	Wait & scan / Wait & scan	NA	NA	NA	NA	NA
3	7	F/ twenties	28	6.0	MQ, 7 mm, sella right, no CSI	DA 12 months, surgery (2x)	No	MQ, equivocal findings, suspected sella right, 7 mm, CSI (Knosp 1); dyn: possibly left	Localization, extension, multifocality, resectability	2.4	Tracer uptake in suspected remnant sella right dorso-laterally, no uptake elsewhere, CSI?	Possibly/ Moderate	Likely / Low	Yes	Surgery / Surgery	Yes	Uncertain (first surgery confirmative)	Yes	Yes	No
3	8	F/ fifties	194	6.8	NA	DA 72 months	No	GQ, equivocal findings, suspected sella left, 5 mm, CSI (Knosp 1); dyn: possibly right	Localization, multifocality, resectability	5.4	Tracer uptake in suspected remnant left, no uptake elsewhere, no CSI	Possibly / Low	Likely / Low	No	Surgery / Surgery	Dubio us	Confirmative	No	Yes	No, temporary DI
3	10	F/ twenties	134	48	MQ, 16 mm, sella left/right, no CSI	DA 74 months, surgery	No	MQ, equivocal findings, possibly sella left, 4 mm, no CSI	Localization, resectability	5.5	Tracer uptake in suspected remnant left dorsocaudally, but also right ventrocranially, no CSI	Possibly / High	Possibly / High	No	Surgery / Wait & Scan	NA	NA	NA	NA	NA
3	11	F/ thirties	107	19	PQ, not assessable	DA 91 months	No	GQ, no visible remnant	Localization, resectability	3.9	Equivocal findings: asymmetrical uptake in sella left	Unlikely / Low	Possibly / Low	No	Surgery / Surgery	Yes	Not confirmative	No	No (PRL=, clini-cally+)	No, temporary DI

											> right, stalk deviation to right: suspected for remnant left side, no CSI									
3	14	F/ thirties	87	4.7	MQ, 20 mm, intra/suprasellar, CSI (Knosp 1 right)	DA 73 months	No	GQ, no visible remnant	Localization, resectability	4.6	Tracer uptake sella right posterolaterally, no CSI	Possibly / Moderate	Likely / Low	No	Surgery / Surgery	Yes	Confirmative	No	Yes	No, SIADH

AI: adrenal insufficiency, CS: cavernous sinus, CSI: cavernous sinus invasions, DA: dopamine agonist, DI: diabetes insipidus, dyn: dynamic view, F: female, GQ: good quality, ICA: internal carotid artery, M: male, MQ: moderate quality, PQ: poor quality, SIADH: syndrome of inappropriate ADH, ULN: upper limit of normal.
