# Supplementary material for: Implementation of functional imaging using 11C-methionine PET-CT co-registered with MRI for advanced surgical planning and decision making in prolactinoma surgery
Source: Pituitary. 2022 May 26;25(4):587–601. doi: 10.1007/s11102-022-01230-2 (PMC9345807; doi:10.1007/s11102-022-01230-2)
Supplement: Supplementary file 3 — Supplementary file3 (PDF 696 KB) [file 11102_2022_1230_MOESM3_ESM.pdf]

Online Resource 3: Supplementary Figures

Implementation of functional imaging using ^{11}C-methionine PET-CT co-registered with MRI for advanced surgical planning and decision making in prolactinoma surgery

Pituitary

Leontine E.H. Bakker*, Marco J.T. Verstegen*, Idris Ghariq, Berit M. Verbist, Pieter J. Schutte, Waiel A. Bashari, Mark C. Kruit, Alberto M. Pereira, Mark Gurnell, Nienke R. Biermasz, Wouter R. van Furth[‡], Lenka M. Pereira Arias-Bouda[‡]

Corresponding author: Leontine E.H. Bakker, Leiden University Medical Center, Leiden, The Netherlands, Department of Medicine, Division of Endocrinology and Center for Endocrine Tumors Leiden (CETL), Pituitary Center, l.e.h.bakker@lumc.nl

Supplementary Figure 1

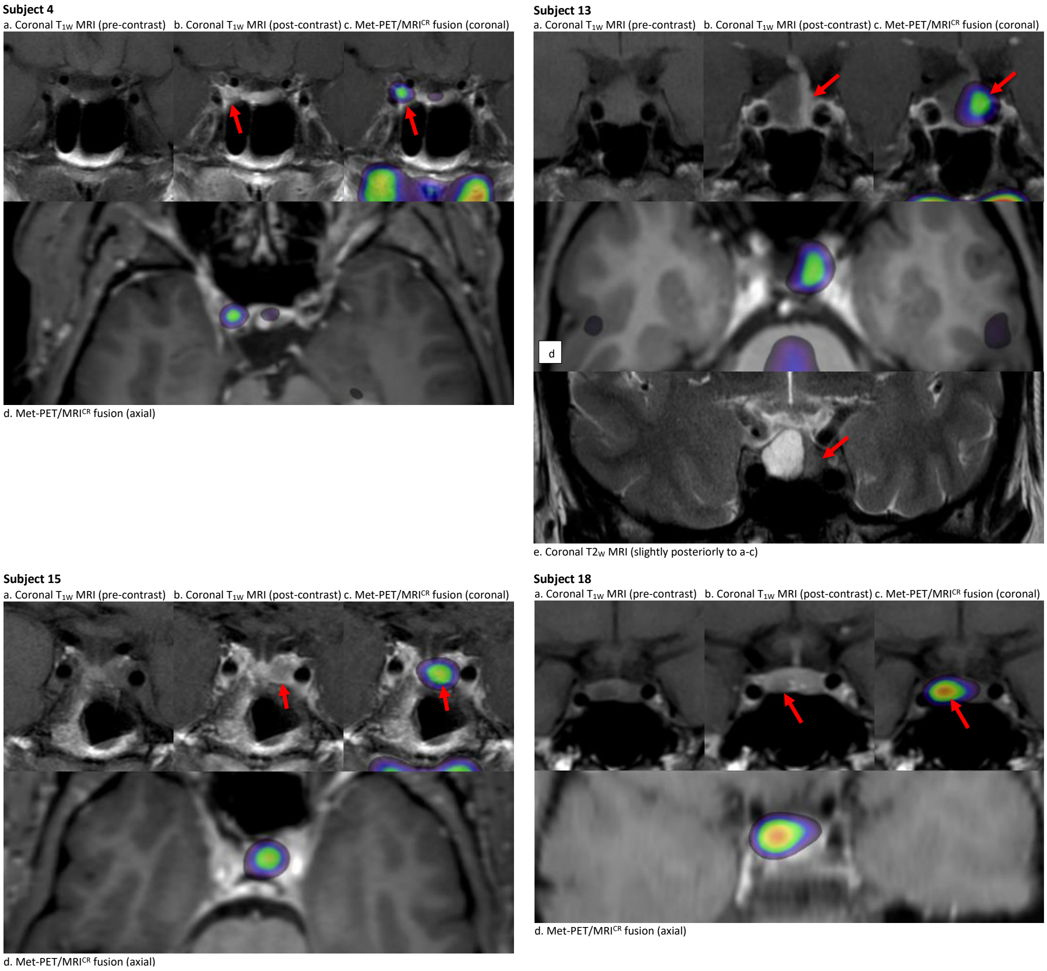

Fig. 1 Imaging in group 1: Met-PET/MRI^{CR} for confirmation (subjects 4, 13, 15 and 18). See Online Resource 1 for a detailed description per subject. T1-weighted coronal MRI pre- and post-gadolinium views and Met-PET/MRI^{CR} coronal and axial views are shown. Arrows denote the suspected site of the adenoma. Met-PET/MRI^{CR}: ^{11}C-methionine positron emission tomography co-registered with magnetic resonance imaging.

Supplementary Figure 2

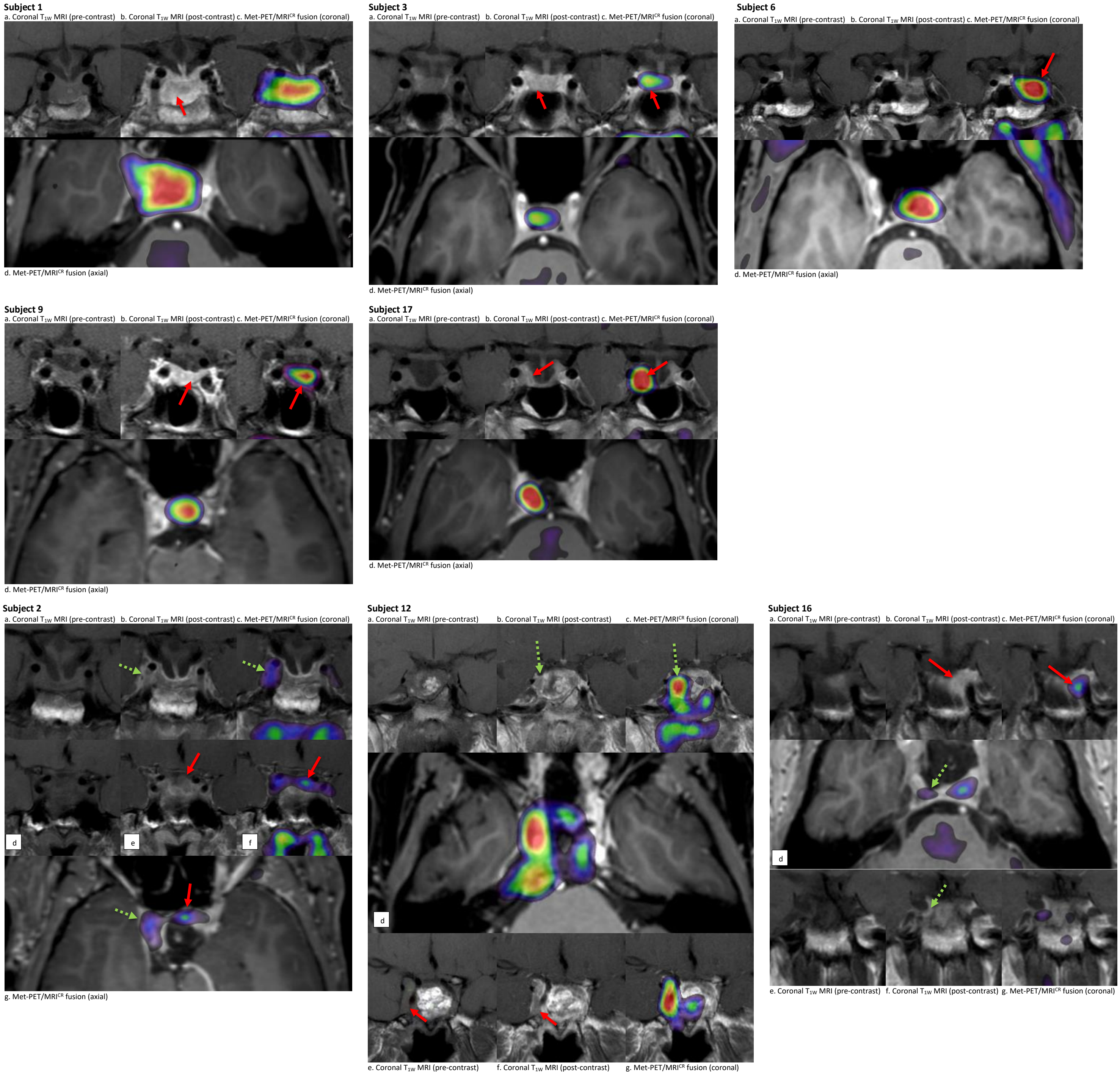

Fig. 2 Imaging in group 2: Met-PET/MRI^{CR} for additional information (subjects 1, 2, 3, 6, 9, 12, 16, 17, and 18). See Online Resource 1 for a detailed description per subject. T1-weighted coronal MRI pre- and post-gadolinium views and Met-PET/MRI^{CR} coronal and axial views are shown. Arrows denote the suspected site of the adenoma. Met-PET/MRI^{CR}: ^{11}C-methionine positron emission tomography co-registered with magnetic resonance imaging.

Supplementary Figure 3

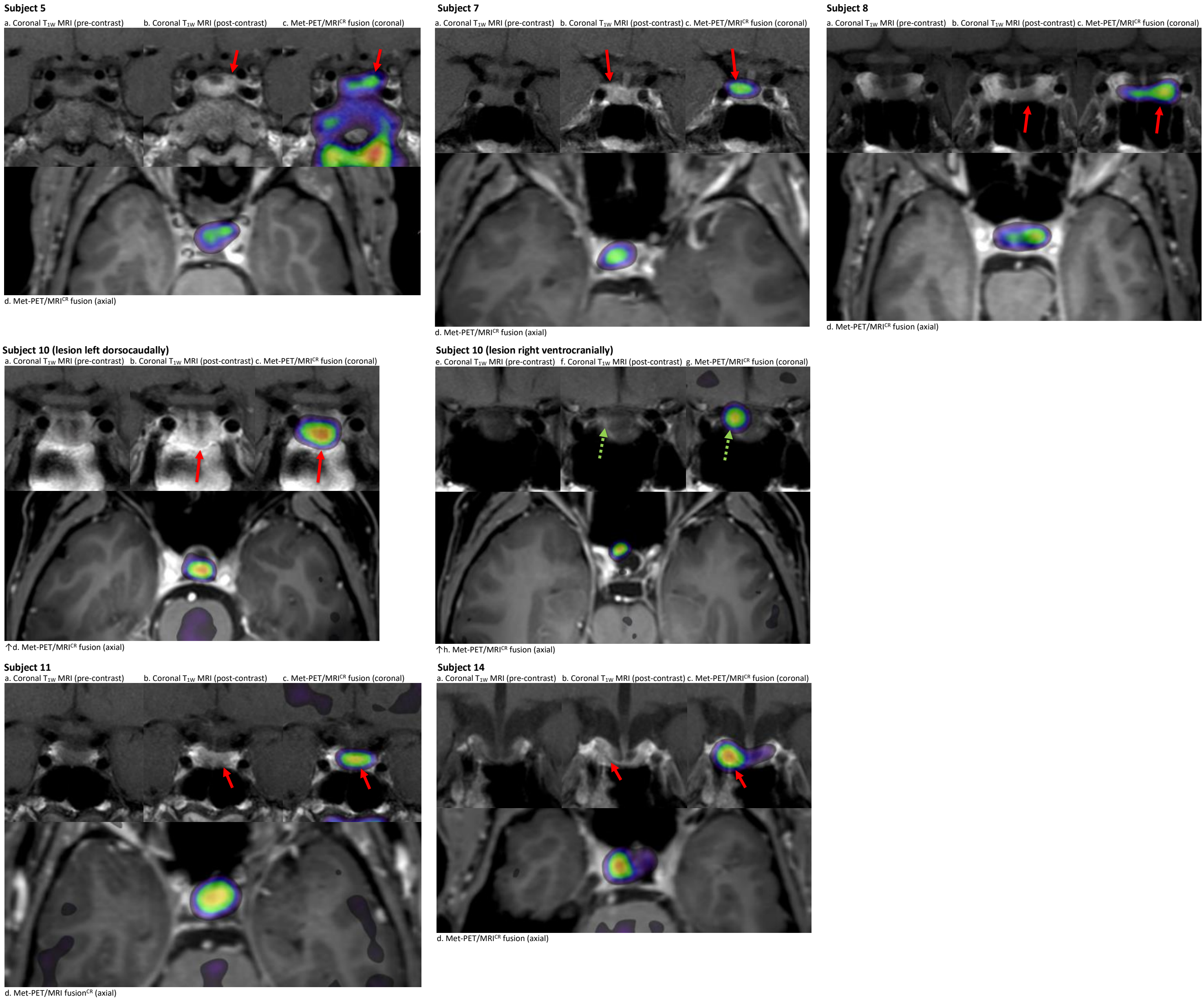

Fig. 3 Imaging in group 3: Met-PET/MRI^{CR} for diagnosis (subjects 5, 7, 8, 10, 11, 14). See Online Resource 1 for a detailed description per subject. T1-weighted coronal MRI pre- and post-gadolinium views and Met-PET/MRI^{CR} coronal and axial views are shown. Arrows denote the suspected site of the adenoma. Met-PET/MRI^{CR}: ^{11}C-methionine positron emission tomography co-registered with magnetic resonance imaging.
